# Supplementary material for: UPP1 promotes lung adenocarcinoma progression through the induction of an immunosuppressive microenvironment
Source: Nat Commun. 2024 Feb 8;15:1200. doi: 10.1038/s41467-024-45340-w (PMC10853547; doi:10.1038/s41467-024-45340-w)
Supplement: Supplementary file 1 — Supplementary information [file 41467_2024_45340_MOESM1_ESM.pdf]

UPP1 promotes lung adenocarcinoma progression through the induction of an immunosuppressive microenvironment

Yin Li[1,#], Manling Jiang[2,#], Ling Aye[3,#] , Li Luo[2,#], Yong Zhang[4], Fengkai Xu[1], Yongqi Wei[1], Dan Peng[2], Xiang He[2], Jie Gu[1], Xiaofang Yu[5], Guoping Li[2,*], Di Ge[1,*], Chunlai Lu[1,*]

[1]Department of Thoracic Surgery, Zhongshan Hospital, Fudan University, Shanghai 200032, China
[2]Laboratory of Allergy and Precision Medicine, Chengdu Institute of Respiratory Health, Affiliated Hospital of Southwest Jiaotong University, The Third People's Hospital of Chengdu, Chengdu 610031, Sichuan, China
[3]Shanghai Medical College, Fudan University, Shanghai, 200032, China
[4]Department of Pulmonary and Critical Care Medicine, Zhongshan Hospital, Fudan University, Shanghai 200032, China
[5]Department of Nephrology, Zhongshan Hospital, Fudan University, Shanghai 200032, China
[#]These authors contributed equally
[*]Corresponding author

Supplementary Figures

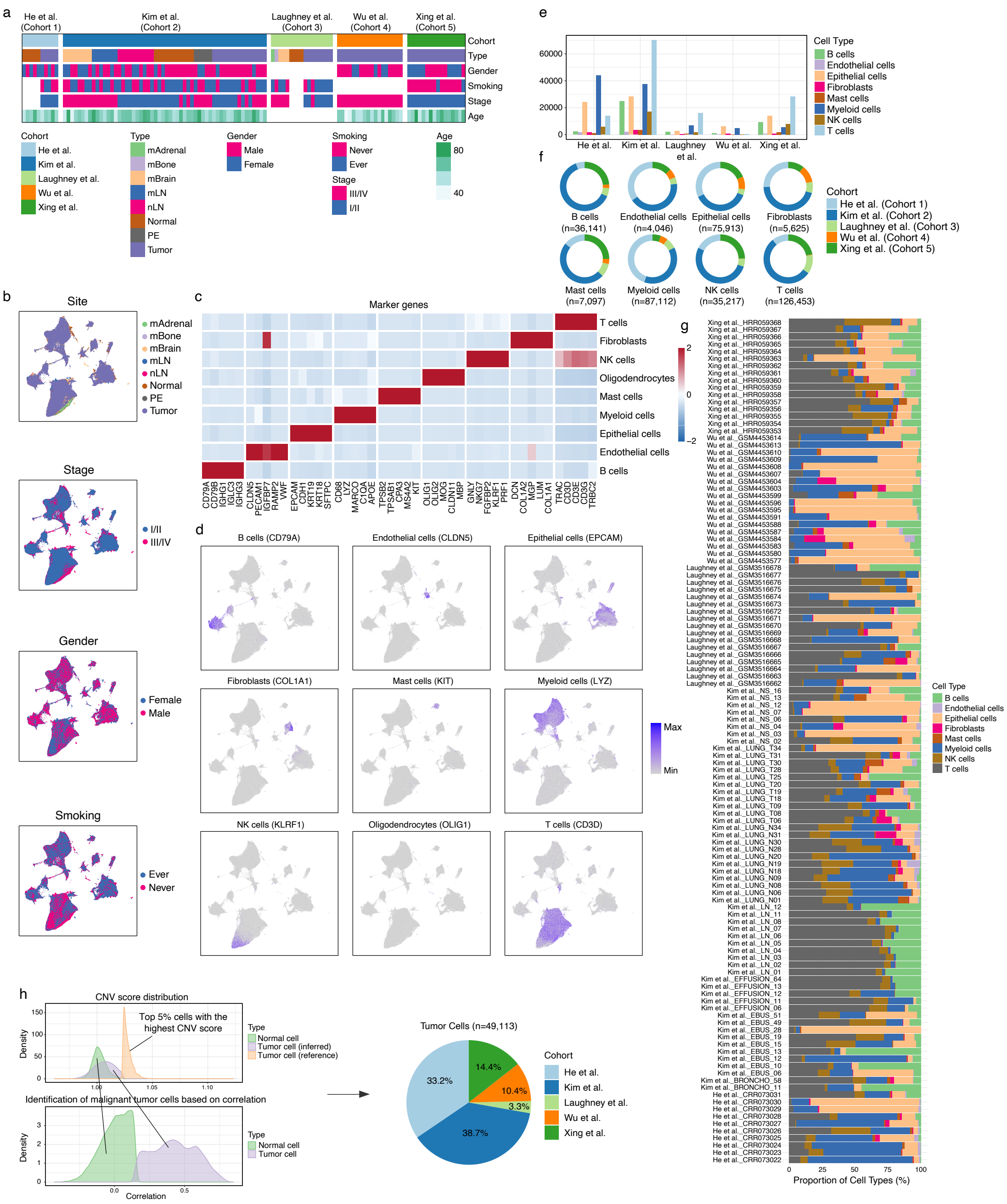

Supplementary Figure 1. Detailed clinical information of LUAD scRNA-seq cohorts, and cell clustering and identification.

a. Clinical information of each LUAD scRNA-seq cohort included in this study. Cohort 1 (10 samples, 88,754 cells)(1), Cohort 2 (56 samples, 181,108 cells)(2), Cohort 3 (17 samples, 29,109 cells)(3), Cohort 4 (18 samples, 12,828 cells)(4), Cohort 5 (16 samples, 65,775 cells)(5). A total of five LUAD scRNA-seq cohorts were enrolled, encompassing samples from mAdrenal (n=1), mBone (n=1), mBrain (n=11), mLN (n=7), nLN (n=10), Normal (n=20), PE (n=5), and Tumor

(n=62). LN, lymph nodes; m, metastatic; PE, pleural effusion. White indicates that the original data was not provided. **b.** The UMAP plots colored by tumor site, clinical stage, mutation status, gender, and smoking status. Cells are evenly distributed. Their distribution is not biased by these different clinical indicators. **c.** Heatmap showing the scaled expression levels of marker genes in the major cell populations. **d.** The UMAP plots showing expression of canonical marker genes of major cell populations, including T cells (CD3D), NK cells (KLFR1), B cells (CD79A), Myeloid cells (LYZ), fibroblasts (COL1A1), mast cells (KIT), endothelial cells (VWF), epithelial cells (EPCAM), and oligodendrocytes (OLIG1). **e.** The number of cells identified in each LUAD scRNA-seq cohort, categorized by cell types. B cells (cells=36,141), Endothelial cells (cells=4,046), Epithelial cells (cells=75,913), Fibroblasts (cells=5,625), Mast cells (cells=7,097), Myeloid cells (cells=87,112), NK cells (cells=35,217), T cells (cells=126,453). **f.** The composition ratios of different LUAD scRNA-seq cohorts within the major cell populations. Each cell type contains samples from different cohorts, demonstrating the effectiveness of data integration. **g.** The proportion of different major cell populations identified in each sample. **h.** Identification of tumor cells based on inferCNV, along with the proportion of tumor cells (n=49,113) identified in each sample. Source data are provided as a Source Data file.

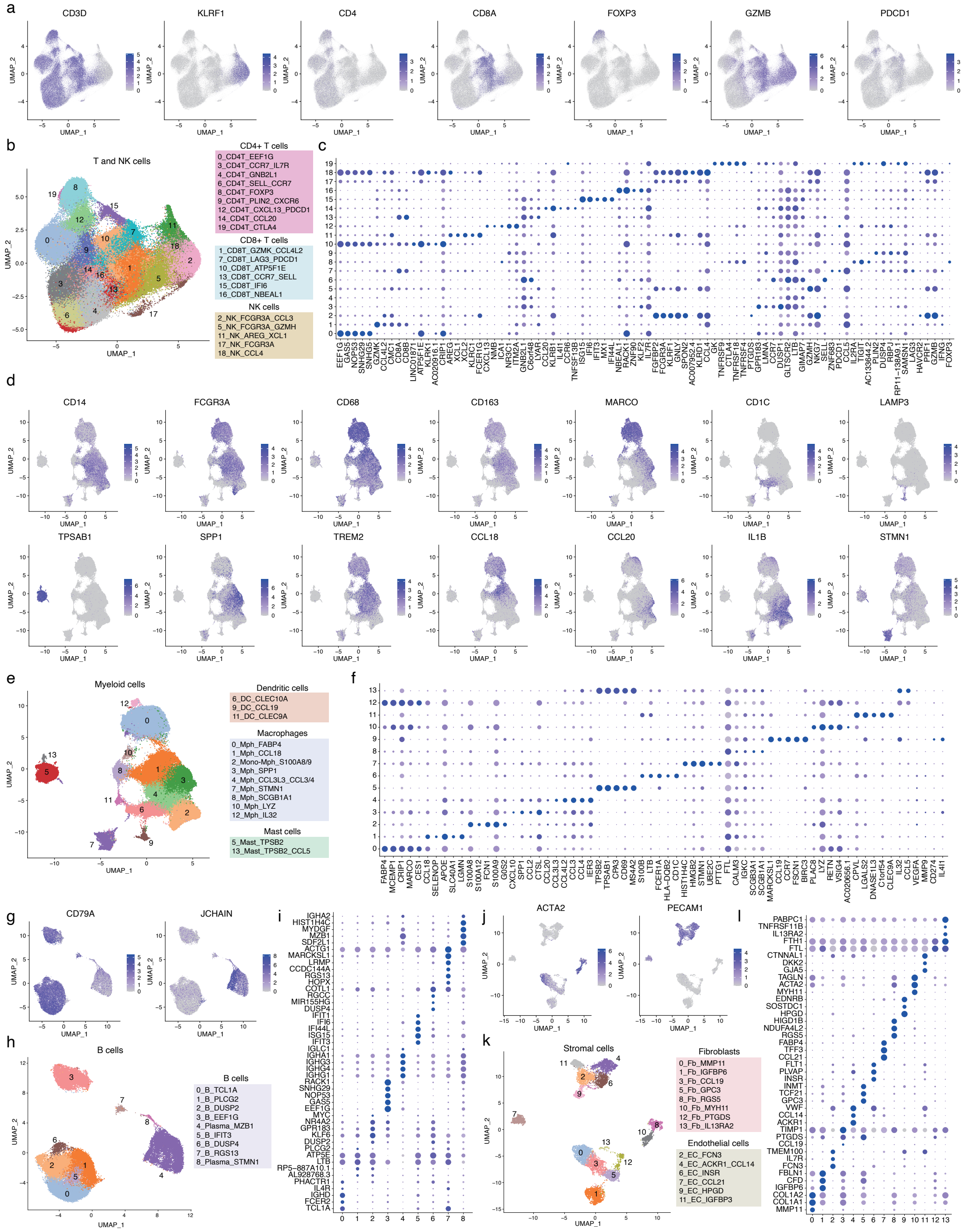

Supplementary Figure 2. Clustering of immune cells and stromal cells.

a. The UMAP plots showing the expression of canonical marker genes for T and NK cells (n=161,670). **b.** The UMAP plots showing the clustering of T and NK cells. Cell clusters are labeled following the "cluster_celltype_marker" format. 9 sub-populations of CD4+ T cells, 6 sub-populations of CD8+ T cells, and 5 sub-populations of NK cells were identified. **c.** Dot plot showing the expression of marker genes for each sub-population in (**b**). **d.** The UMAP plots showing the

expression of canonical marker genes for myeloid cells (n=94,209). **e.** The UMAP plots showing the clustering of myeloid cells. 3 sub-populations of dendritic cells (DC), 9 sub-populations of macrophages (Mph), and 2 sub-populations of mast cells (Mast) were identified. **f.** Dot plot showing the expression of marker genes for each sub-population in (**e**). **g.** The UMAP plots showing the expression of canonical marker genes for B cells (n=36,141). **h.** 9 sub-populations of B cells were identified. **i.** Dot plot showing the expression of marker genes for each sub-population in (**h**). **j.** The UMAP plots showing the expression of canonical marker genes for stromal cells (n=81,538). **k.** 8 sub-populations of fibroblasts (Fb) and 6 sub-populations of endothelial cells (EC) were identified. **l.** Dot plot showing the expression of marker genes for each sub-population in (**k**). Source data are provided as a Source Data file.

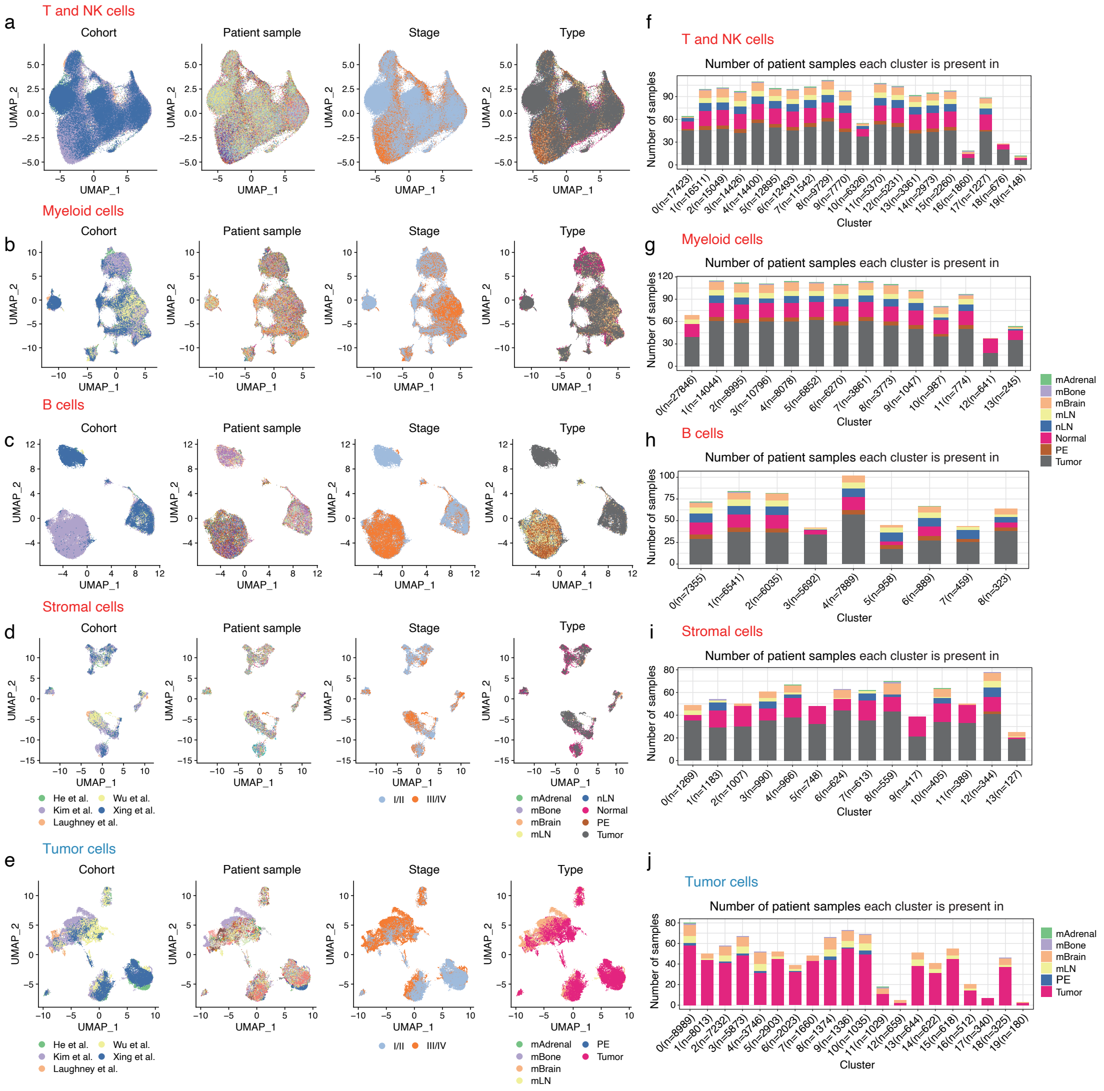

Supplementary Figure 3. Distribution of sub-populations.

a-e. The UMAP plots showing the distribution of T and NK cells (n=161,670) **(a)**, myeloid cells (n=94,209) **(b)**, B cells (n=36,141) **(c)**, stromal cells (n=81,538) **(d)**, and tumor cells (n=49,113) **(e)**, colored by LUAD scRNA-seq cohort, patient sample, stage, and sample type. Dots represent individual cells. **f-j.** The number of cells in each T and NK cell sub-population **(f)**, myeloid cell sub-population **(g)**, B cell sub-population **(h)**, stromal cell sub-population **(i)**, and tumor cell sub-population **(j)**, and their composition by sample source. The x-axis represents the number of cells in the sub-population and the y-axis represents how many samples this sub-population appears in. Source data are provided as a Source Data file.

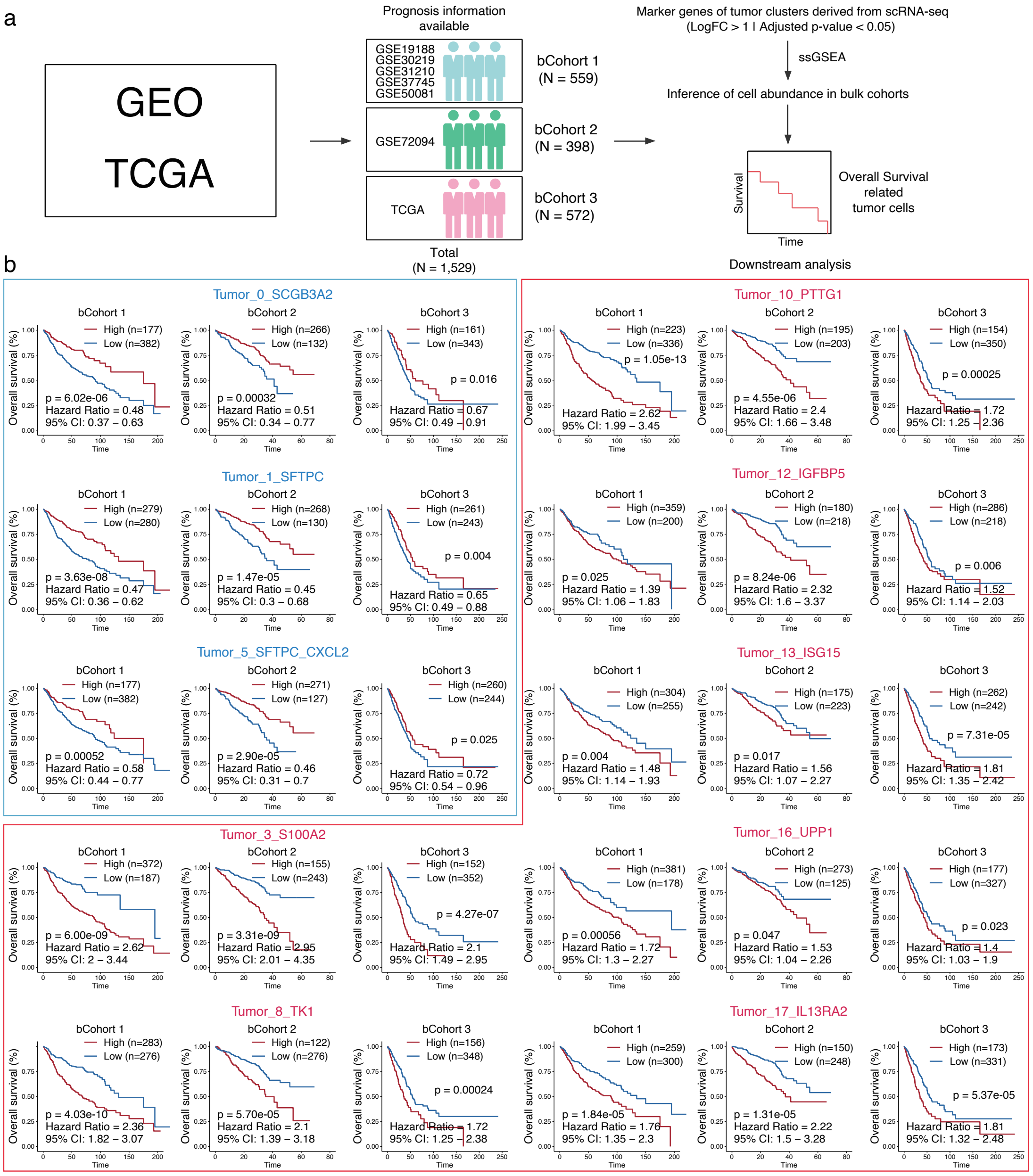

Supplementary Figure 4. Exploring the prognostic implications of tumor cell sub-populations using LUAD bulk cohorts.

a. Study workflow. Gene expression data of LUAD patients with complete clinical prognosis information (overall survival) from the GEO and TCGA databases were collected and integrated. The gene expression data of LUAD patients were divided into three independent cohorts. The first cohort (bCohort1) consists of data from 5 GEO datasets with a total of 559 patients(6-10). The second cohort (bCohort2) is derived from a single independent GEO dataset, including 398 patients(11). The third cohort (bCohort3) is based on TCGA dataset and includes 572 patients(12). Subsequently, the marker genes (Log2FC >1 and adjusted p-value < 0.05) for the tumor cell sub-populations identified from scRNA-seq data were used to calculate the abundances of these tumor cell sub-populations in bulk cohorts using ssGSEA method. The prognosis analysis was conducted by correlating these cell abundances with the overall survival (OS) of the patients. **b.** The survival curves for tumor cell sub-populations that were significantly associated with patient prognosis in all of the three bulk cohorts ($p < 0.05$). The survival curves in blue boxes represent sub-populations that were correlated with better prognosis, while the survival curves in red boxes represent sub-populations that were relatively associated with worse prognosis. Statistical analysis was performed using log-rank tests. Source data are provided as a Source Data file.

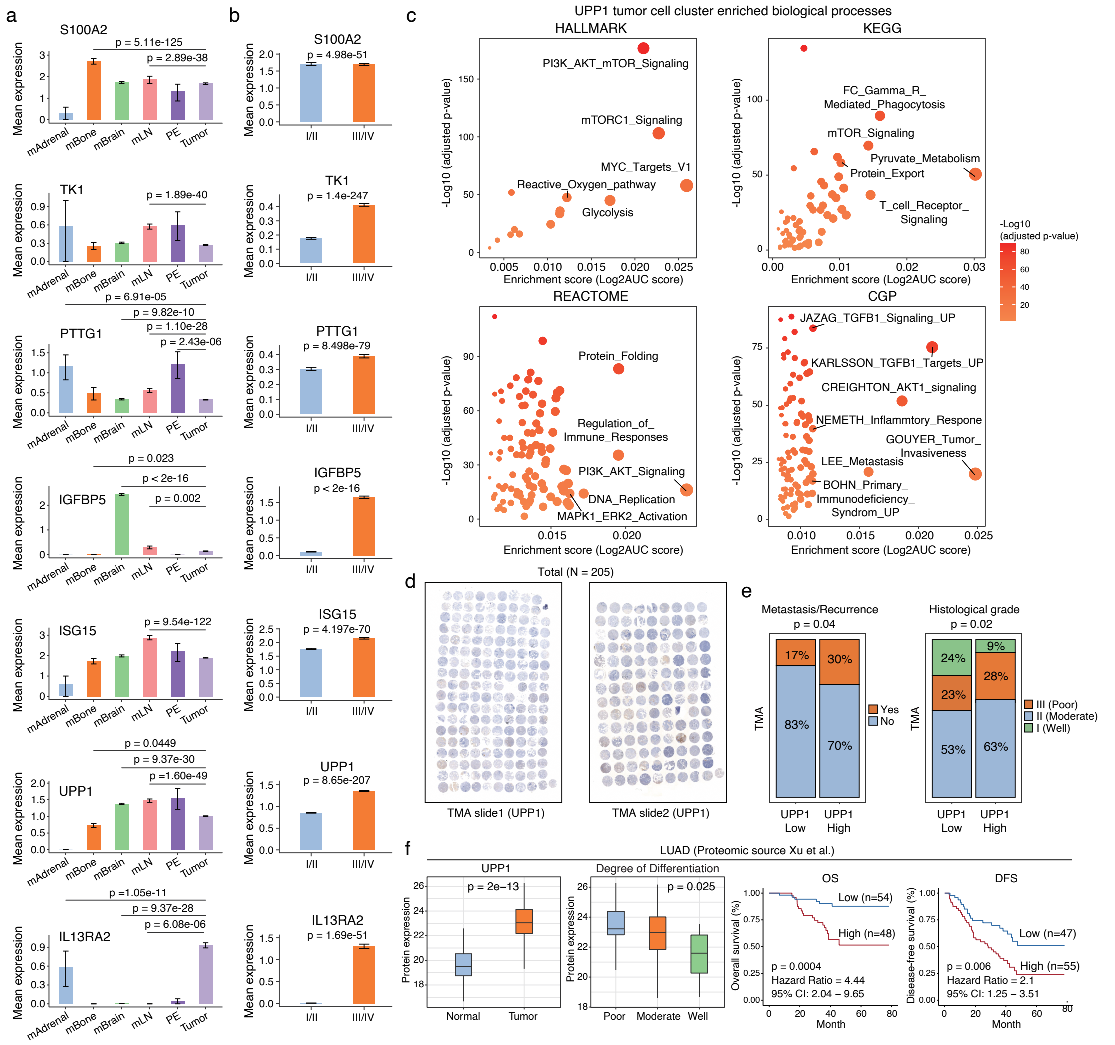

Supplementary Figure 5. Expression of the marker genes of tumor cell sub-populations associated with poor patient prognosis, functional enrichment analysis of UPP1^{high} tumor cells, and the correlation between UPP1 expression and patient prognosis.

a. Comparison of the expression of the marker genes of tumor cell sub-populations associated with poor patient prognosis in different tumor sites (cells=49,113). mAdrenal (n=1, cells=4), mBone (n=1, cells=334), mBrain (n=11, cells=10,449), mLN (n=7, cells= 1,407), PE (n=5, cells=35), and Tumor (n=62, cells= 36,884). Data were represented as mean ± SD. Statistical analysis was conducted using the two-tailed Wilcoxon rank-sum test. **b.** Comparison of the expression of the marker genes of tumor cell sub-populations associated with poor patient prognosis in different stages. Data were represented as mean ± SD. Statistical analysis was conducted using the two-tailed Wilcoxon rank-sum test. **c.** Functional enrichment analysis of UPP1^{high} tumor cells. Enrichment analysis was performed respectively using HALLMARK, KEGG, REACTOME, and Chemical and Genetic

Perturbations (CGP) gene sets. Multiple hypothesis testing was performed using Bonferroni correction, and terms with adjusted p-values less than 0.05 were considered significant. Terms of interest are displayed. **d.** IHC staining of UPP1 in our TMA cohort (n=205). **e.** The relationship between UPP1 expression and metastasis/recurrence, as well as disease grade (n=205). Statistical analysis was conducted using two-tailed chi-squared tests. **f.** Analysis of UPP1 protein expression levels in the proteomic dataset from Xu et al. From left to right(13): Comparison of UPP1 expression between tumor samples (n=102) and normal sample (n=40), statistical analysis was conducted using the two-tailed Wilcoxon rank-sum test; Comparison of UPP1 expression with degree of differentiation (n=102), statistical analysis was conducted using the two-tailed Kruskal-Wallis test, for boxplots, central line shows median, box limits indicate upper and lower quartiles, and whiskers extend 1.5 times the interquartile range; Association between UPP1 and patient overall survival (OS, n=102) and disease-free survival (DFS, n=102), statistical analysis was performed using log-rank tests. Source data are provided as a Source Data file.

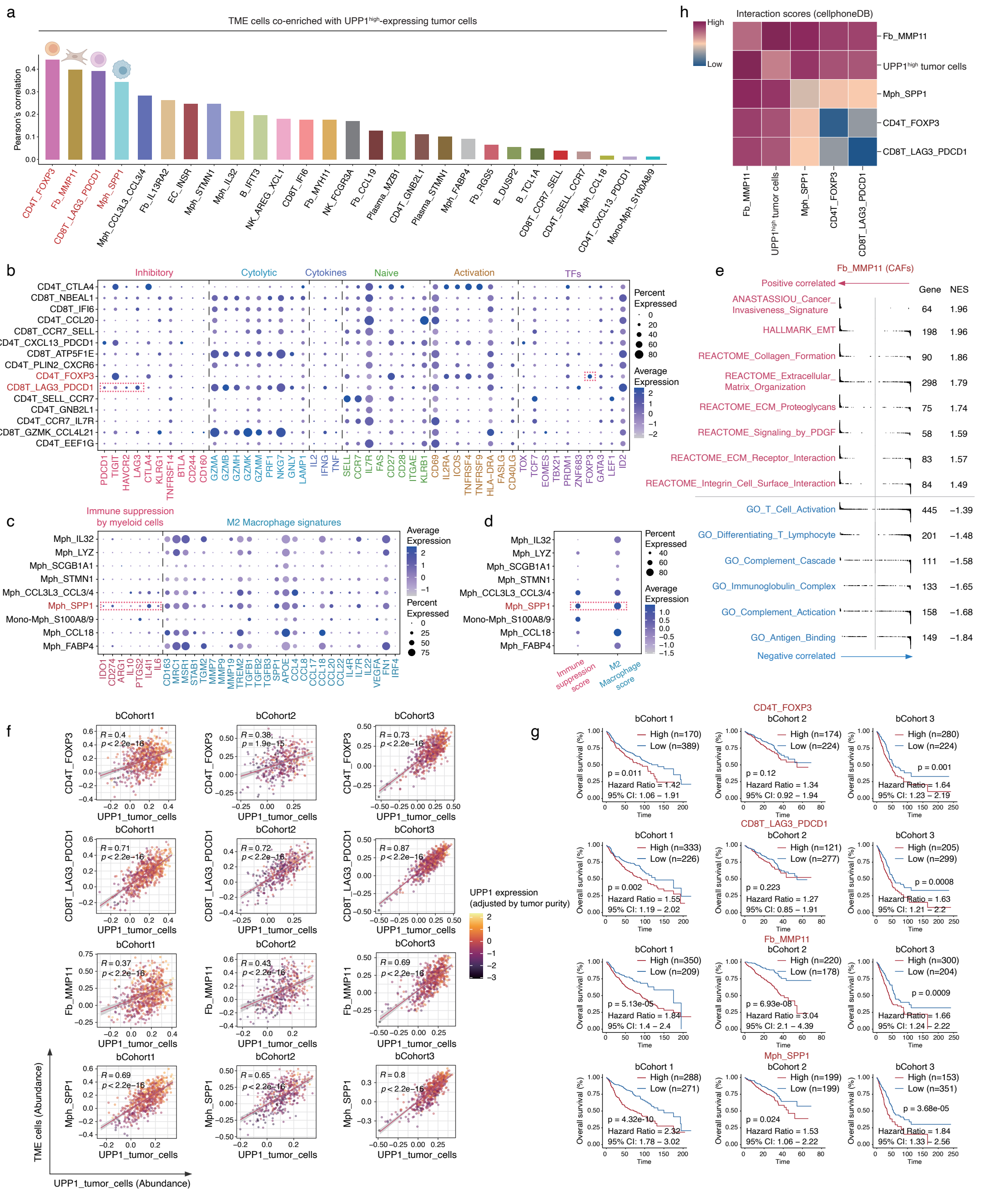

Supplementary Figure 6. Exploring the interactions between UPP1^{high} tumor cells and TME cell populations.

a. The co-enrichment patterns of UPP1^{high} tumor cells with other cell populations on the basis of correlations of cell clusters. Correlations greater than 0.3 are considered as significant. Statistical analysis was conducted using two-tailed Pearson's correlation. From this analysis, UPP1^{high} tumor cells were found significantly correlated with CD4T_FOXP3 (FOXP3+ regulatory T cells), CD8T_LAG3_PDCD1 (LAG3+PDCD1+ CD8+ exhausted T cells), Mph_SPP1 (SPP1+ M2 macrophages), and Fb_MMP11 (MMP11+ CAFs) cell clusters. In this analysis, UPP1^{high} expressing

tumor cells were defined as tumor cells with UPP1 expression levels higher than the median UPP1 expression. **b.** Dot plot showing the expression of canonical functional marker genes of T cells to confirm the features of CD4T_FOXP3 and CD8T_LAG3_PDCD1 cell clusters. **c.** Dot plot showing the expression of canonical functional marker genes of immunosuppressive M2-like macrophages to confirm the features of Mph_SPP1 cell cluster. **d.** Assessing the functional features of Mph_SPP1 cell cluster. The immune-suppression-by-myeloid-cell and M2-macrophage scores were calculated based on the mean expression of genes in (**c**). The Mph_SPP1 cell cluster exhibited prominent features of immunosuppressive M2-like macrophages. **e.** Functional enrichment analysis of the Fb_MMP11 cell cluster confirmed its characteristics as cancer-associated fibroblasts (CAFs). Functional enrichment analysis was conducted using GSVA method (adjusted p-value < 0.05). **f.** Inference the abundances of CD4T_FOXP3, CD8T_LAG3_PDCD1, Mph_SPP1, Fb_MMP11, and $UPP1^{high}$ tumor cells in the bulk cohorts based on the marker genes (adjusted p-value < 0.05) of these cell clusters using the ssGSEA method, and their correlations with $UPP1^{high}$ tumor cell. Statistical analysis was conducted using two-tailed Pearson's correlation. **g.** The overall survival curves of CD4T_FOXP3, CD8T_LAG3_PDCD1, Mph_SPP1, and Fb_MMP11 cell clusters in the bulk cohorts. Statistical analysis was performed using log-rank tests (bCohort1, n=559; bCohort2, n=398; bCohort3, n=572). **h.** Validation of the inter-cellular interactions between CD4T_FOXP3, CD8T_LAG3_PDCD1, Mph_SPP1, Fb_MMP11, and $UPP1^{high}$ tumor cells using CellphoneDB analysis. Source data are provided as a Source Data file.

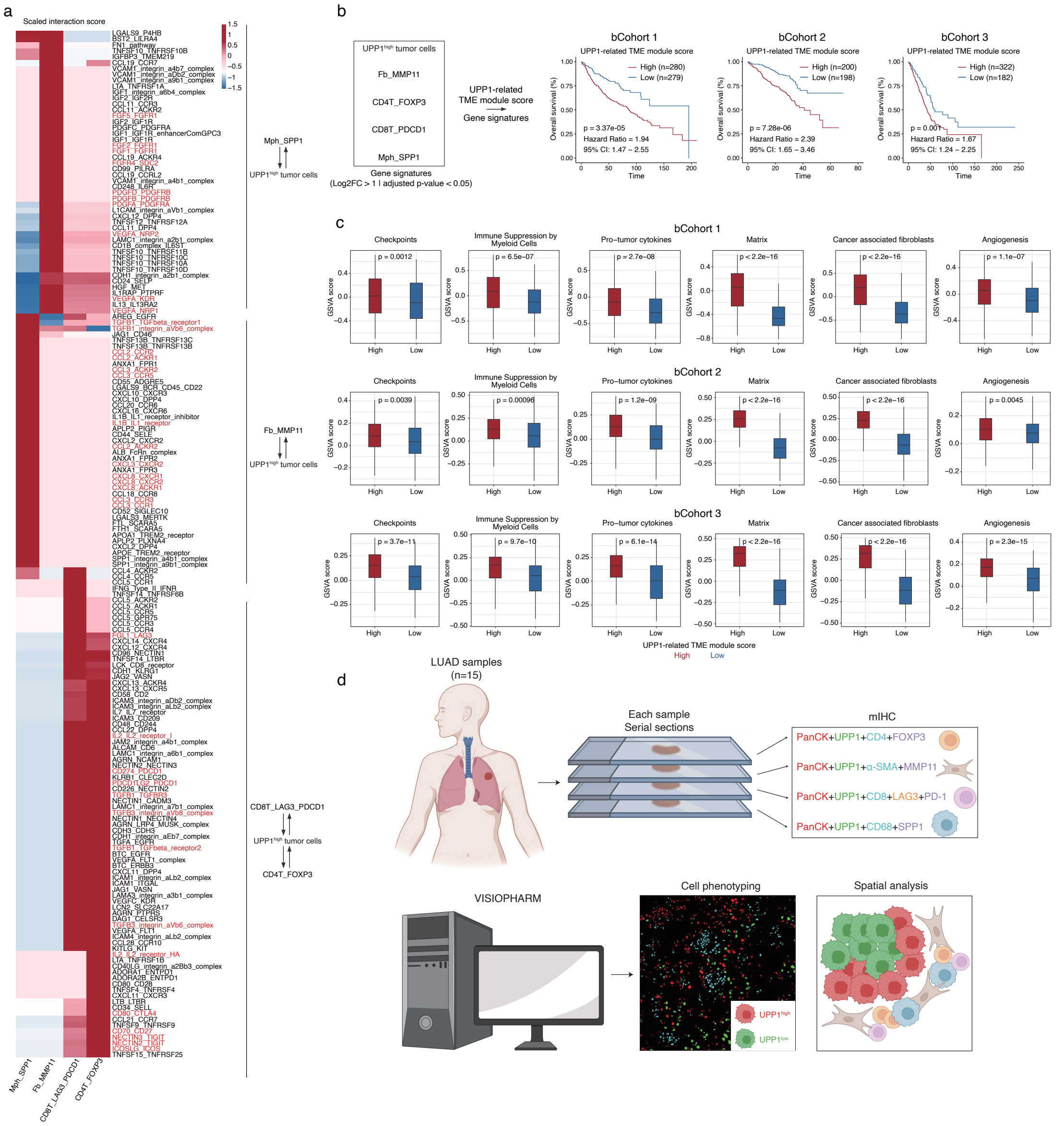

Supplementary Figure 7. The correlation of UPP1^{high} tumor cells with immunosuppressive TME.

a. The ligand-receptor cell-cell interactions between UPP1^{high} tumor cells and CD4T_FOXP3, CD8T_LAG3_PDCD1, Mph_SPP1, and Fb_MMP11 cells using CellphoneDB analysis. The ligand-receptor pairs of interest are highlighted in red. **b.** Establishment of the UPP1-related TME module based on the marker genes (Log2FC > 1 and adjusted p-value < 0.05) of these five cell populations. The UPP1-related TME module score for each patient within the bulk datasets was calculated using the GSVA method, and its correlation with patients' OS was analyzed. Statistical analysis was performed using log-rank tests (bCohort1, n=559; bCohort2, n=398; bCohort3, n=572). **c.** Comparison of the TME features between patients in the high and low UPP1-related TME module

groups. Statistical analysis was conducted using the two-tailed Wilcoxon rank-sum test. For boxplots, central line shows median, box limits indicate upper and lower quartiles, and whiskers extend 1.5 times the interquartile range (bCohort1, n=559; bCohort2, n=398; bCohort3, n=572). **d.** Multiplex immunofluorescence staining (mIF) on LUAD patient samples (n=15) to validate the correlations between UPP1^{high} tumor cells and related four groups of cell populations, created with BioRender.com. For each tumor specimen, we prepared four serial sections and performed staining of on each to validate the interactions between UPP1^{high} tumor cells and FOXP3+CD4+ T cells, MMP11+ fibroblasts, LAG3+PDCD1+CD8+ T cells, and SPP1+ macrophages. Unbiased phenotypic identification and spatial analysis were performed using AI-based Visiopharm software. Source data are provided as a Source Data file.

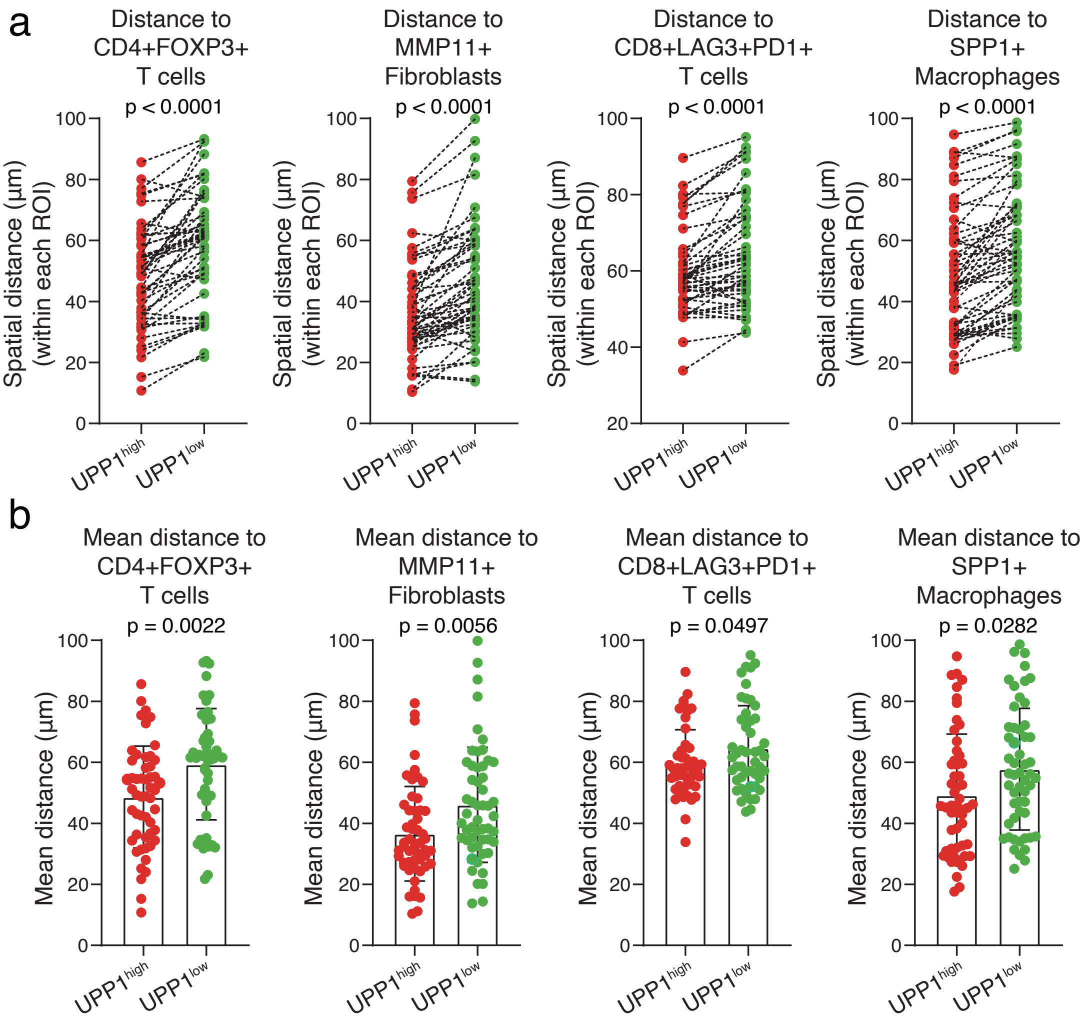

Supplementary Figure 8. Spatial analysis of UPP1^{high} tumor cells.

a. For each image, different regions of interest (ROIs) were selected. Within each ROI, the average spatial distance between UPP1^{high} tumor cells or UPP1^{low} tumor cells with the corresponding cell populations were respectively calculated. Red represents the spatial distances between UPP1^{high} tumor cells and the corresponding cell populations, while green represents the spatial distances between UPP1^{low} tumor cells and the corresponding cell populations. FOXP3+CD4+ T cells (number of ROIs=52), MMP11+ fibroblasts (number of ROIs=53), LAG3+PDCD1+CD8+ T cells (number of ROIs=48), and SPP1+ macrophages (number of ROIs=54). Statistical analysis was conducted using the two-tailed paired student's t-test. **b.** Comparison of the average spatial distances. Data were represented as mean ± SD. Statistical analysis was conducted using the two-tailed student's t-test. Source data are provided as a Source Data file.

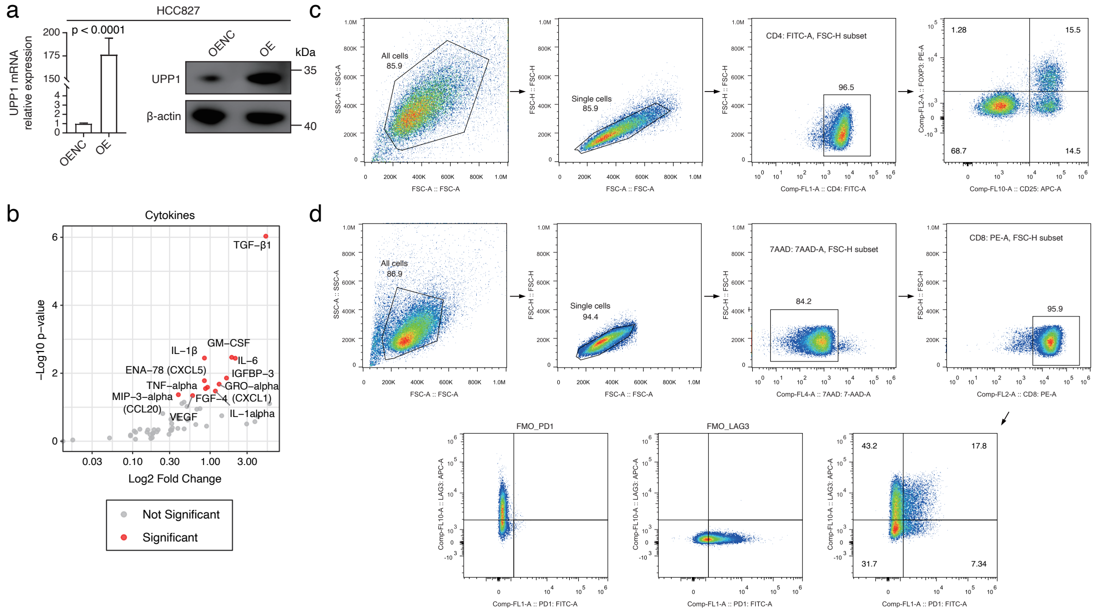

Supplementary Figure 9. Co-culture experiment.

a. Verification of the effectiveness of UPP1 overexpression in HCC827 tumor cells using both RT-qPCR and Western blotting (n=4). RT-qPCR data are presented as mean ± SEM. Statistical analysis was conducted using the two-tailed student's t-test. n denotes biologically independent samples. **b.** Evaluation of cytokine differences between the UPP1-overexpressing cell group and the control group (n=3). Cytokines with significant variations were highlighted. Statistical analysis was conducted using the two-tailed student's t-test. n denotes biologically independent samples. **c.** Gating strategy for the analysis of CD4+ T cells in Figure 3e. **d.** Gating strategy for the analysis of

CD8+ T cells in Figure 3f. Source data are provided as a Source Data file.

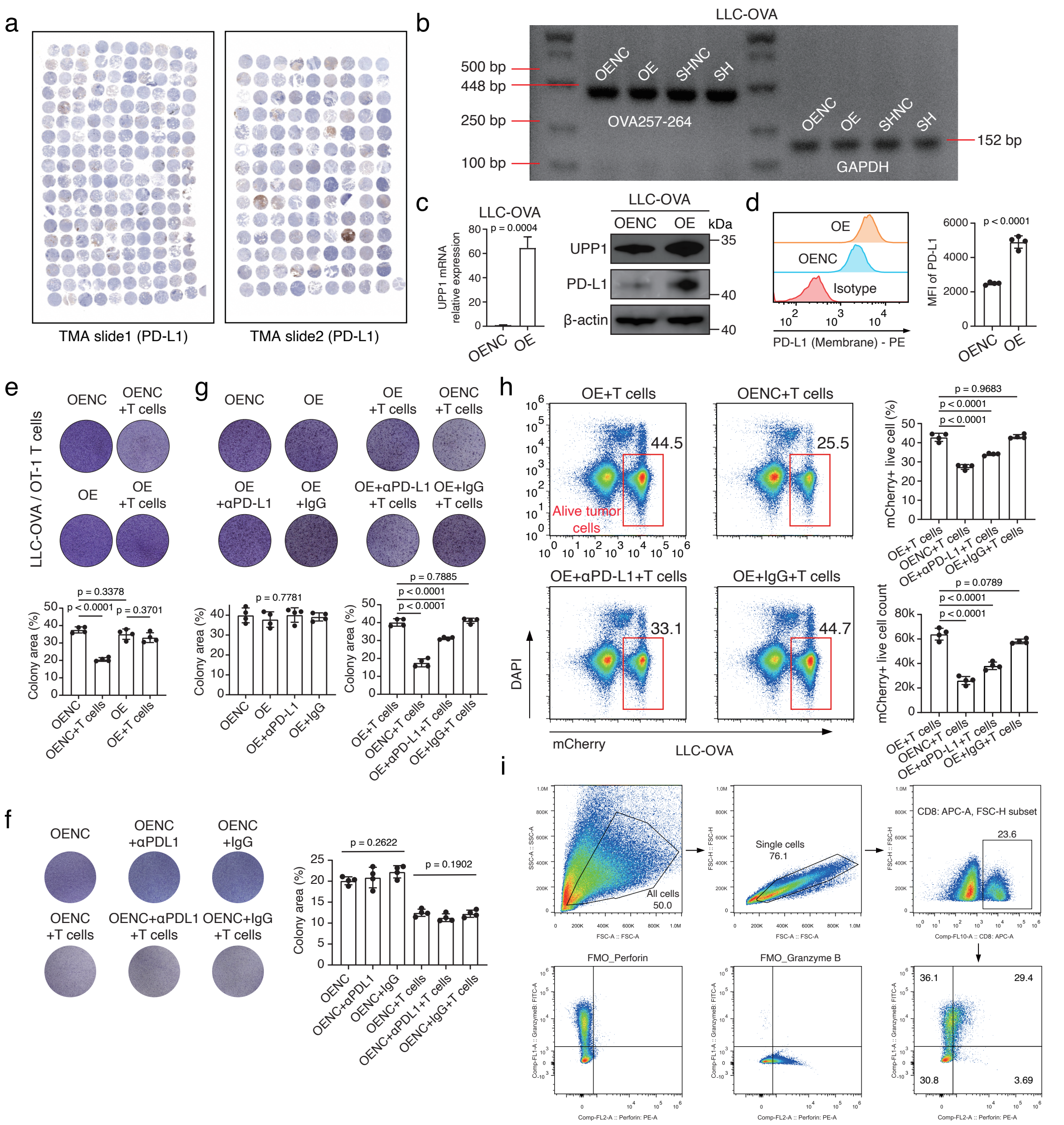

Supplementary Figure 10. Upregulation of PD-L1 induced by UPP1 overexpression in tumor cells inhibited the killing ability of CD8+ T cells.

a. IHC staining of PD-L1 in our TMA cohort (n=205). **b.** Verification of OVA expression on LLC-OVA tumor cells via PCR following UPP1 overexpression or knockdown revealed no impact of UPP1 alterations on OVA expression. **c.** Left: Verification of the effectiveness of UPP1 overexpression (OE) in LLC-OVA tumor cells using RT-qPCR (n=4). Data are presented as mean ± SEM. Statistical analysis was conducted using the two-tailed student's t-test. Right: Western blot analysis of PD-L1 expression in LLC-OVA tumor cells with UPP1 overexpression (OE) (n=3). **d.**

Flow cytometry analysis of PD-L1 expression on the cell surface of LLC-OVA tumor cells with UPP1 overexpression (n=4). Statistical analysis was conducted using the two-tailed student's t-test. MFI, mean fluorescence intensity. **e.** Crystal violet staining of surviving LLC-OVA tumor cells following co-culture with OT-1 CD8+ T cells (n=4). Statistical analysis was conducted using one-way ANOVA with multiple comparisons. **f.** Crystal violet staining of surviving LLC-OVA tumor cells following co-culture with OT-1 CD8+ T cells (n=4). Statistical analysis was conducted using one-way ANOVA. **g.** Crystal violet staining of surviving LLC-OVA tumor cells after co-culture. Left: Confirmation that PD-L1 antibodies (αPD-L1) and isotype control IgG had no influence on tumor cell viability during the co-culture period. Right: crystal violet staining following the co-culture of UPP1-overexpressed LLC-OVA tumor cells with OT-1 CD8+ T cells in the presence of PD-L1 antibodies (n=4). Statistical analysis was conducted using one-way ANOVA with multiple comparisons. **h.** Flow cytometry analysis of the count and percent of surviving tumor cells following the co-culture of UPP1-overexpressed LLC-OVA tumor cells with OT-1 CD8+ T cells in the presence of PD-L1 antibodies. LLC-OVA tumor cells were labeled with mCherry (n=4). Statistical analysis was conducted using one-way ANOVA with multiple comparisons. **i.** Gating strategy for the analysis of OT-1 CD8+ T cells in Figure 4i. n denotes biologically independent samples. Data are presented as mean ± SD in **d**, **e**, **f**, **g**, and **h.** Source data are provided as a Source Data file.

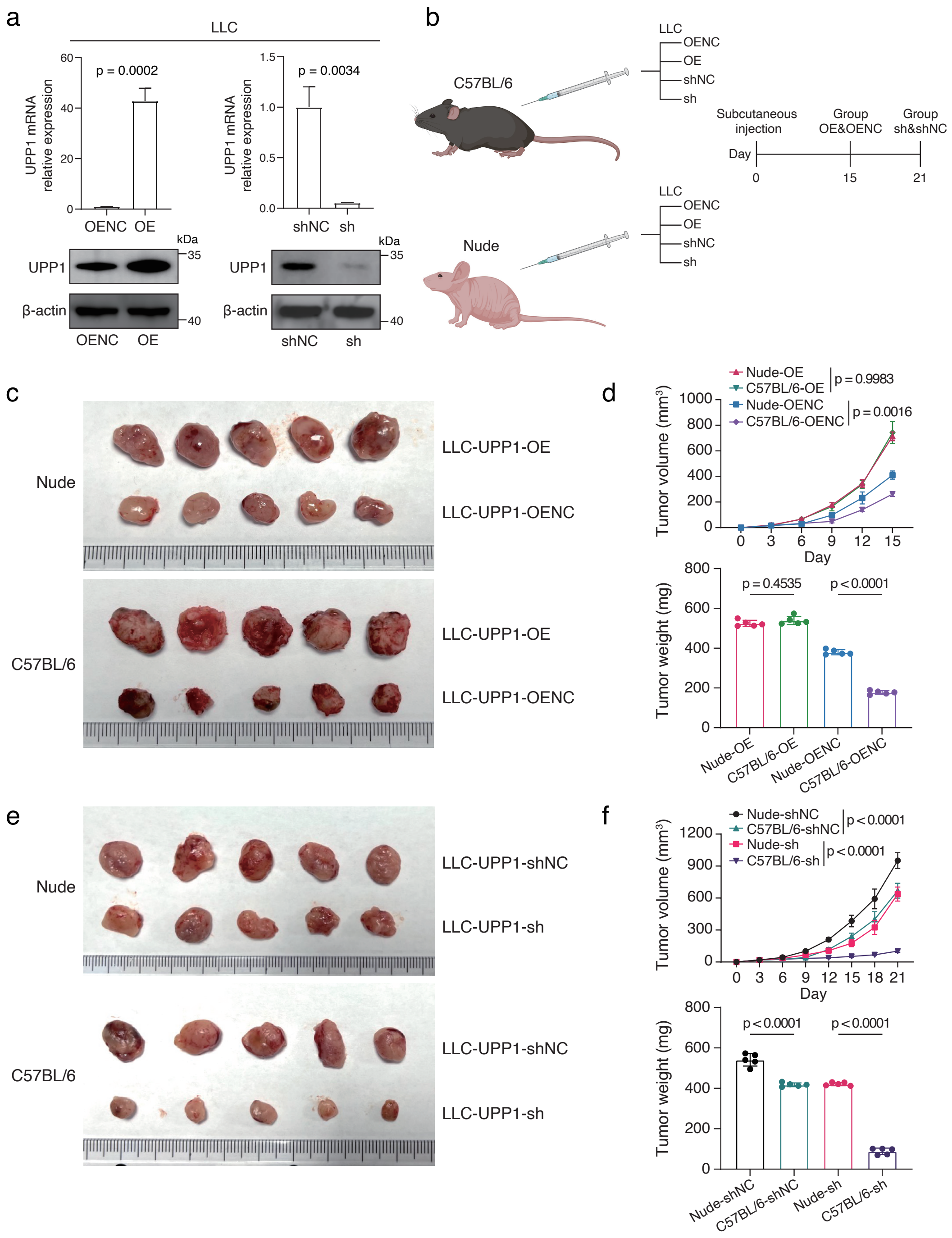

Supplementary Figure 11. Verification of the association between UPP1 and tumor immunity through mice experiments.

a. Verification of the effectiveness of UPP1 overexpression and UPP1 knockdown in LLC tumor cells using both RT-qPCR and Western blotting (n=4). RT-qPCR data are presented as mean ± SEM. Statistical analysis was conducted using the two-tailed student's t-test. n denotes biologically independent samples. **b.** Experimental workflow, created with BioRender.com. UPP1-overexpressing tumor cells (LLC-UPP1-OE), UPP1-downregulated tumor cells (LLC-UPP1-sh),

and their respective controls (LLC-UPP1-OENC/ LLC-UPP1-shNC) were subcutaneously implanted into both C57BL/6 mice and nude mice, respectively. **c.** Tumors harvested from mice bearing LLC-UPP1-OE tumor cells and LLC-UPP1-OENC tumor cells (n=5). Top, nude mice; Bottom, C57BL/6 mice. n denotes biologically independent samples. **d.** Top, tumor growth curves from (**c**) (n = 5); Bottom, Tumor weight at day 15 from (**c**) (n = 5). Data are presented as mean ± SD. Statistical analysis was performed using two-way ANOVA with multiple comparisons. n denotes biologically independent samples. **e.** Tumors harvested from mice bearing LLC-UPP1-sh tumor cells and LLC-UPP1-shNC tumor cells (n=5). Top, nude mice; Bottom, C57BL/6 mice. n denotes biologically independent samples. **f.** Top, tumor growth curves from (**f**) (n = 5); Bottom, Tumor weight at day 21 from (**f**) (n = 5). Data are presented as mean ± SD. Statistical analysis was performed using two-way ANOVA with multiple comparisons. n denotes biologically independent samples. Source data are provided as a Source Data file.

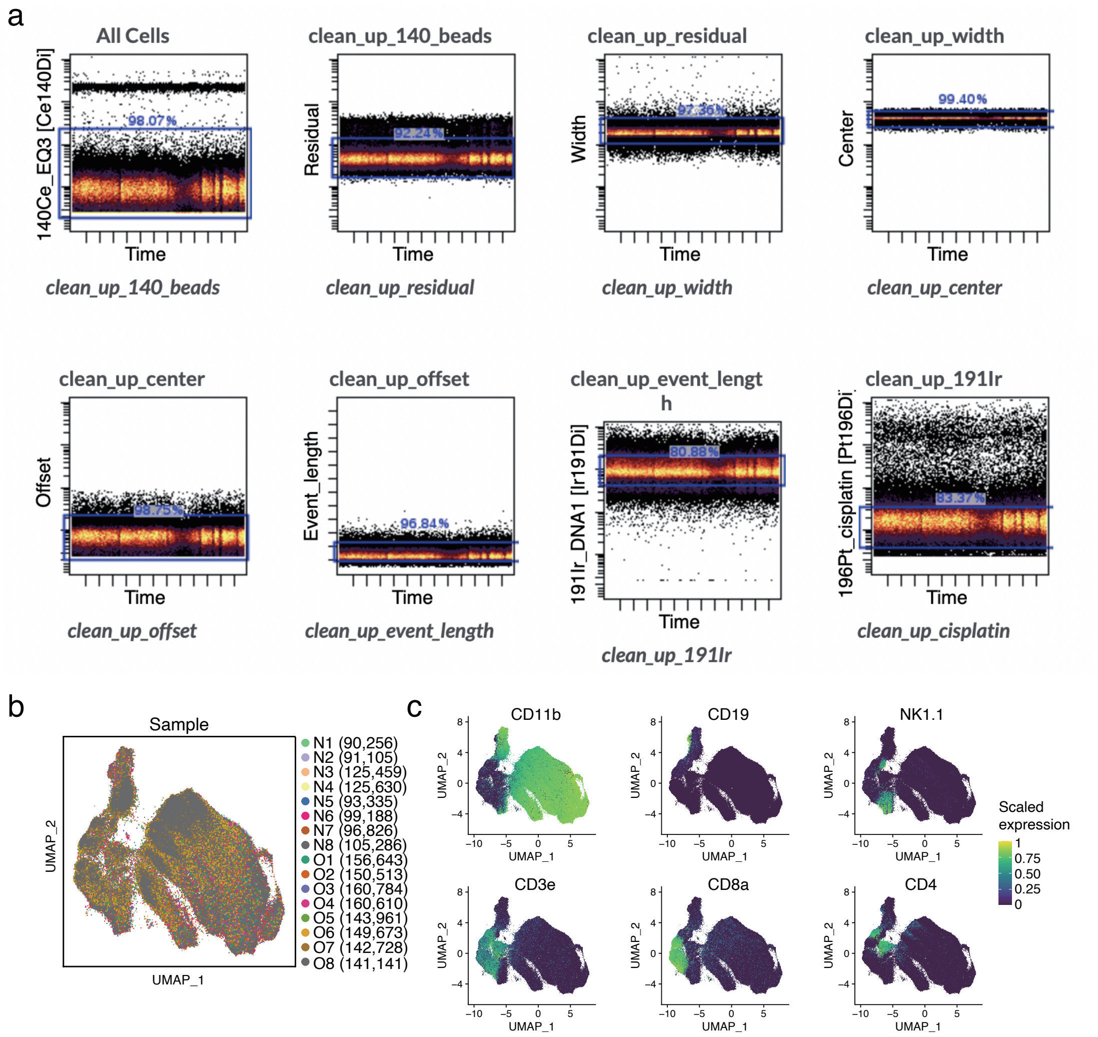

Supplementary Figure 12. CyTOF analysis of the characteristics of TME.

a. Quality control of CyTOF data using Cytobank (http://www.cytobank.cn). This process involved the removal of beads (140_beads), dead cells (cisplatin), doublets (191Ir), as well as adjustments for residual, width, center, offset, and event length. **b.** The UMAP plot showing the sample distributions. Dots represent individual cells, and colors represent different samples. The number represents the number of events (cells) obtained in each sample. N, UPP1-OENC; O, UPP1-OE. **c.** The UMAP plot showing the expression of canonical markers, including CD11b, CD19, NK1.1, CD3e, CD4, and CD8a.

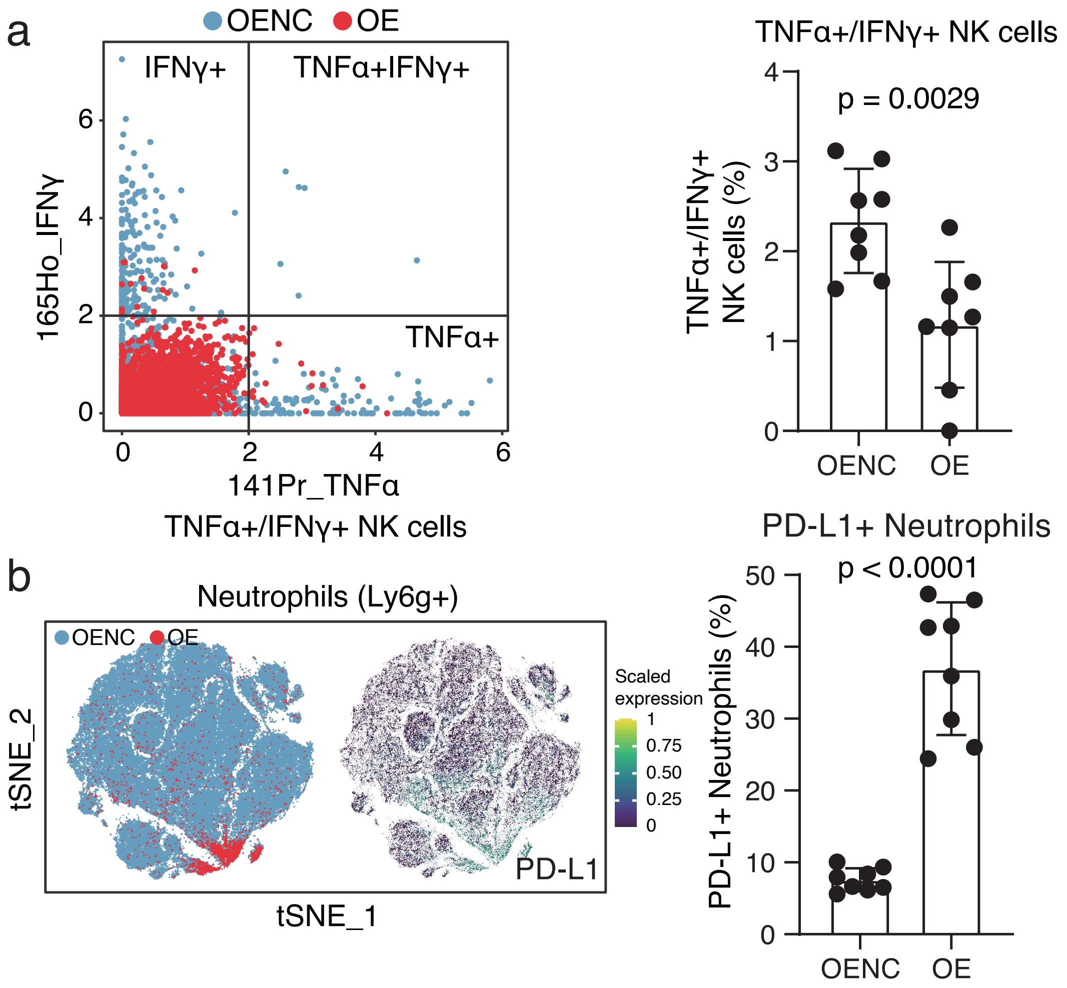

Supplementary Figure 13. NK cells and neutrophils in CyTOF analysis.

a. Comparison of the proportion of TNFα+/IFNγ+ NK cells/total NK cells between the UPP1-OE and UPP1-OENC groups (n = 8). Data are presented as mean ± SD. Statistical analysis was conducted using the two-tailed student's t-test. **b.** Comparison of the proportion of PD-L1+ neutrophils (Ly6G+)/total neutrophils between the UPP1-OE and UPP1-OENC groups (n = 8). Data are presented as mean ± SD. Statistical analysis was conducted using the two-tailed student's t-test. Source data are provided as a Source Data file.

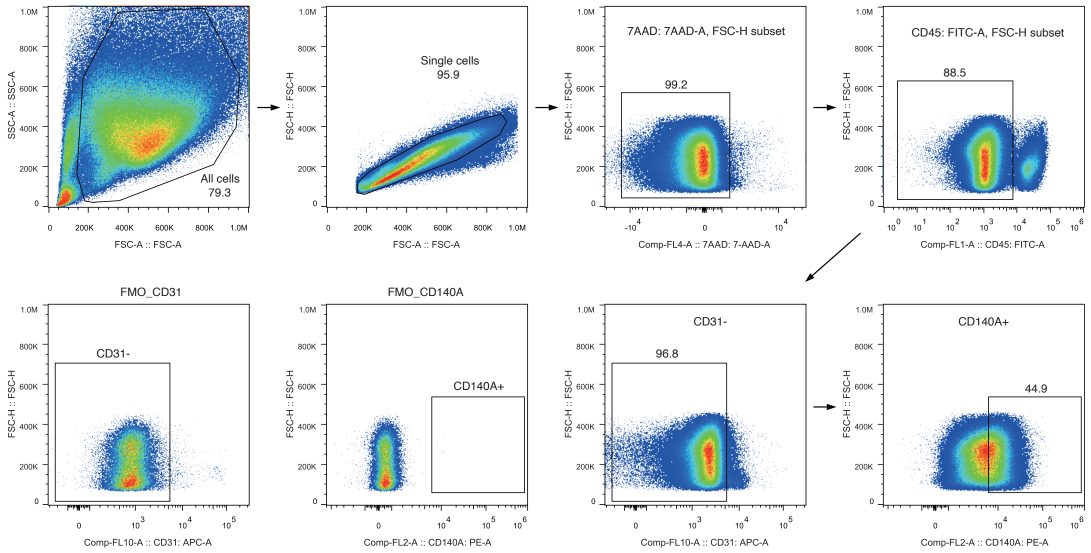

Supplementary Figure 14. Gating strategy for the analysis of fibroblasts in Figure 5l.

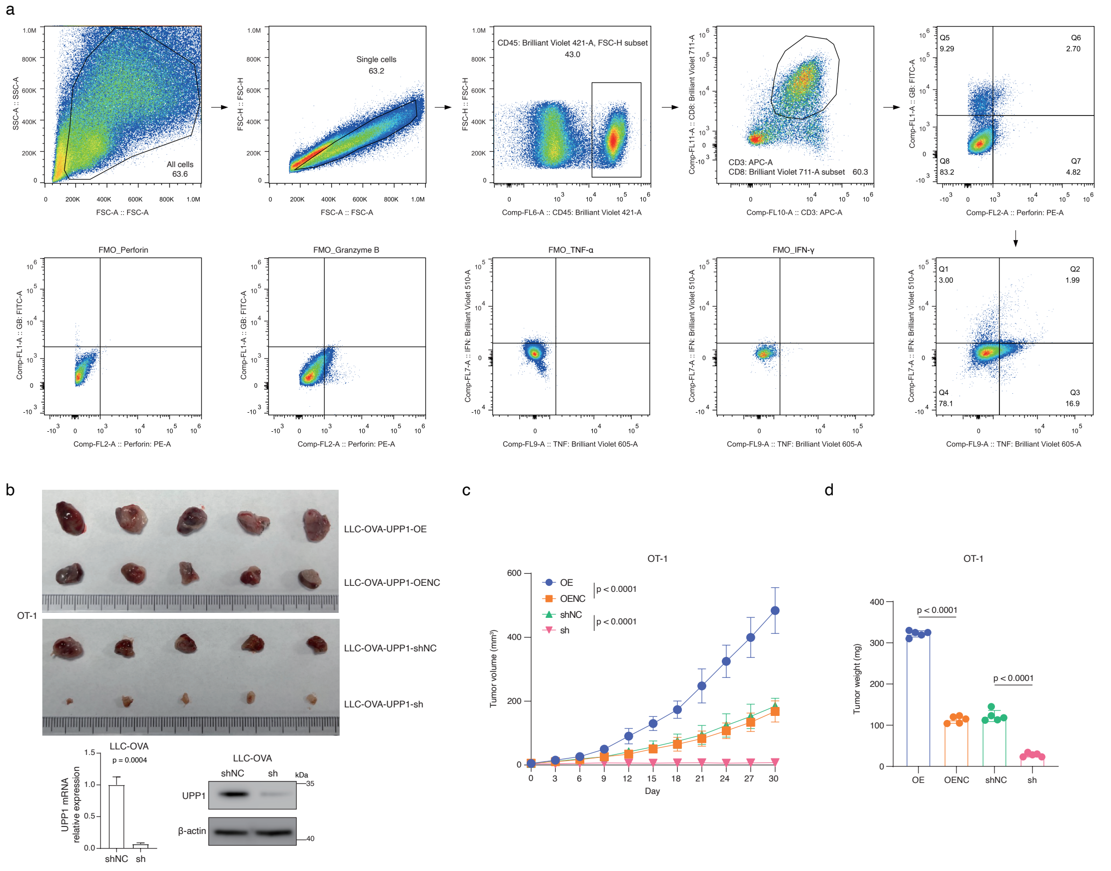

Supplementary Figure 15. The changes of UPP1 expression on tumor progression in OT-1 mice.

a. Gating strategy for the analysis of CD8+ T cells in Figure 6e-g. **b.** Top: Tumors harvested from

OT-1 mice (n=5) bearing LLC-OVA-UPP1-OE tumor cells, LLC-OVA-UPP1-OENC tumor cells, LLC-OVA-UPP1-shNC tumor cells, and LLC-OVA-UPP1-sh tumor cells. Bottom: Verification of the effectiveness of UPP1 knockdown in LLC-OVA tumor cells using both RT-qPCR and Western blotting (n=4). RT-qPCR data are presented as mean ± SEM. Statistical analysis was conducted using the two-tailed student's t-test. n denotes biologically independent samples. **c.** Tumor growth curves from (**b**) (n = 5). Data are presented as mean ± SD. Statistical analysis was performed using two-way ANOVA with multiple comparisons. n denotes biologically independent samples. **d.** Tumor weight at day 30 from (**b**) (n = 5). Data are presented as mean ± SD. Statistical analysis was performed using one-way ANOVA with multiple comparisons. n denotes biologically independent samples. Source data are provided as a Source Data file.

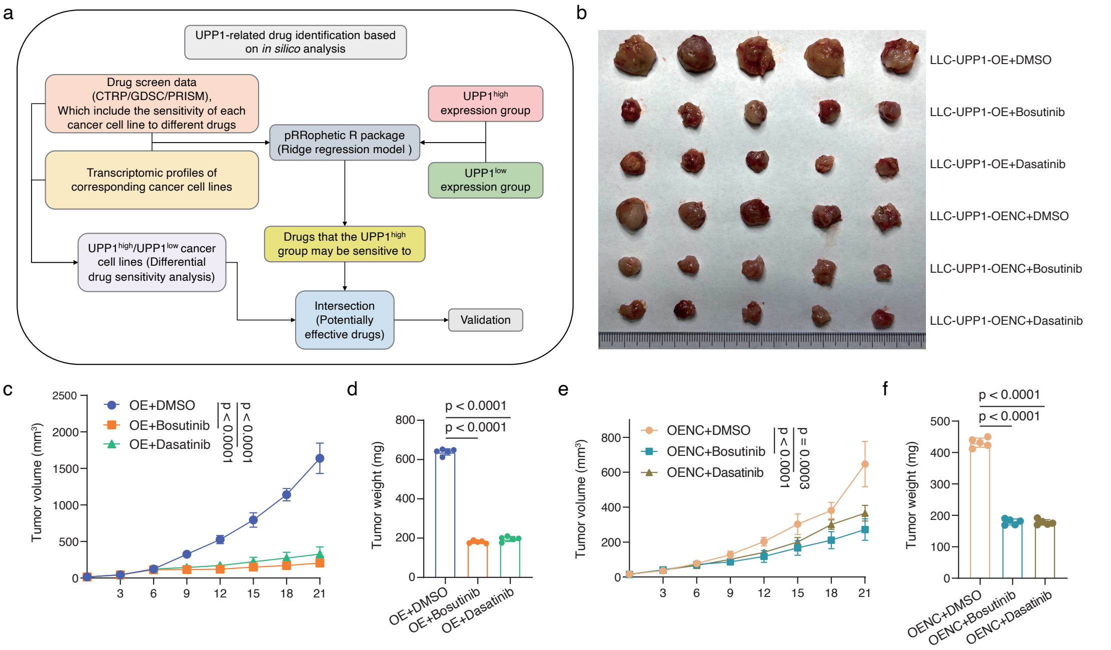

Supplementary Figure 16. Bioinformatics-based drug screening and validation.

a. Detailed study workflow. **b.** Collection of tumors from C57BL/6 mice (n=5) implanted with either LLC-OVA-UPP1-OE or LLC-OVA-UPP1-OENC tumor cells, treated with either Bosutinib or Dasatinib. n denotes biologically independent samples. **c.** Tumor growth curves comparing the comparative efficacy of Bosutinib and Dasatinib in mice bearing LLC-OVA-UPP1-OE tumor cells (n = 5). Data are presented as mean ± SD. Statistical analysis was performed using two-way ANOVA with multiple comparisons. n denotes biologically independent samples. **d.** Tumor weight at day 21

from (C) (n = 5). Data are presented as mean ± SD. Statistical analysis was performed using one-way ANOVA with multiple comparisons n denotes biologically independent samples. **e.** Tumor growth curves comparing the comparative efficacy of Bosutinib and Dasatinib in mice bearing LLC-OVA-UPP1-OENC tumor cells (n = 5). Data are presented as mean ± SD. Statistical analysis was performed using two-way ANOVA with multiple comparisons. n denotes biologically independent samples. **f.** Tumor weight at day 21 from (E) (n = 5). Data are presented as mean ± SD. Statistical analysis was performed using one-way ANOVA with multiple comparisons. n denotes biologically independent samples. Source data are provided as a Source Data file.

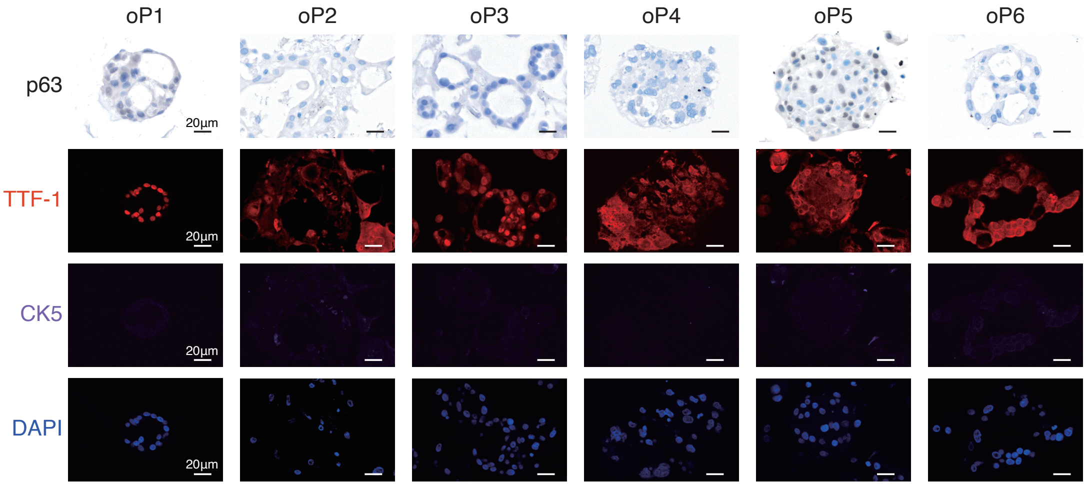

Supplementary Figure 17. Validation of the tumor purity of PDOs based on expression of p63, TTF-1, and CK5.

Supplementary Tables

Supplementary Table 1: Antibodies used for CyTOF analysis.

**Marker**	**Clone**	**Metal**	**Cat No.**
CD11b [MAC1]	M1/70	110Cd	(Fluidigm, 92J001110), 1:100
TCRb	H57-597	143Nd	(Fluidigm, 3143010B), 1:100
CD25	3C7	150Nd	(Fluidigm, 3150002B), 1:100
CD3e	145-2C11	152Sm	(Fluidigm, 3152004B), 1:100
PD-L1	MIH5	153Eu	(Fluidigm, 3153031B), 1:100
PD-1	RMP1-30	159Tb	(Fluidigm, 3159006B), 1:100
CD163	EPR19518	161Dy	(Abcam, ab213612), 1:100
TIM3	RMT3-23	162Dy	(Fluidigm, 3162029B), 1:100
CD8a	53-6.7	168Er	(Fluidigm, 3168003B), 1:200
NK1.1	PK136	170Er	(Fluidigm, 3170002B), 1:100
CD19	6D5	171Yb	(Fluidigm, 92J025171), 1:200
CD4	RM4-5	172Yb	(Fluidigm, 3172003B), 1:200
LAG3	C9B7W	174Yb	(Fluidigm, 3174019B), 1:100
CTLA-4	UC10-4B9	175Lu	(Abcam, ab251599), 1:100
Ly-6G	1A8	195Pt	(Fluidigm, 92J011195), 1:200
CD45	30-F11	89Y	(Fluidigm, 3089005B), 1:200
TNFa	MP6-XT22	141Pr	(Fluidigm, 3141013B), 1:100
IL-4	11B11	155Gd	(Biolegend, 504129), 1:100
IL-10	JES5-16E3	158Gd	(Fluidigm, 3158002B), 1:100
IFNg	XMG1.2	165Ho	(Fluidigm, 3165003B), 1:100
FOXP3	FJK-16s	149Sm	(Invitrogen, 14-5773-82), 1:100

Supplementary Table 2: PCR primers.

RT-qPCR Primers	(5'-3')
GAPDH-Forward	AGGTCGGAGTCAACGGATTTGG
GAPDH-Reverse	TGCCATGGGTGGAATCATATTGG
UPP1-Forward	TGATTGCCCCGTCAGACTTTT
UPP1-Reverse	CACCAACGCACCTGATGAAG
SPP1-Forward	CTCCATTGACTCGAACGACTC
SPP1-Reverse	CAGGTCTGCGAAACTTCTTAGAT
CD274-Forward	AGGGCATTCCAGAAAGATGAGG
CD274-Reverse	TGTATGGGGCGTTCAGCAAA
CD163-Forward	CTTGGGACTTGGACGATGCT
CD163-Reverse	GGTATCTTAAAGGCTCACTGGGT
CCL20-Forward	GTCTGTGTGCGCAAATCCAA
CCL20-Reverse	CCAACCCCAGCAAGGTTCTT
Gapdh-Forward	AAGAAGGTGGTGAAGCAGG
Gapdh-Reverse	GAAGGTGGAAGAGTGGGAGT
Fap-Forward	GTCACCTGATCGGCAATTTGT
Fap-Reverse	CCCCATTCTGAAGGTCGTAGAT
Mmp11-Forward	GGTTTCCACCATCCGAGGAG
Mmp11-Reverse	GTCTGAATCACCCCTGAATGC
Upp1-Forward	ACAGGAACTGAAGCAAAGGAC
Upp1-Reverse	GTTGAAATGGTAGAGCACGTCTT
PCR Primers	(5'-3')
Ova(257-264)-Forward	CACAAGCAATGCCTTTCAGA
Ova(257-264)-Reverse	TACCACCTCTCTGCCTGCTT
Gapdh-Forward	TCATGACCACAGTCCATGCC
Gapdh-Reverse	TCAGCTCTGGGATGACCTTG

Supplementary Reference

1. He D, Wang D, Lu P, Yang N, Xue Z, Zhu X, et al. Single-cell RNA sequencing reveals heterogeneous tumor and immune cell populations in early-stage lung adenocarcinomas harboring EGFR mutations. Oncogene. 2021;40(2):355-68.
2. Kim N, Kim HK, Lee K, Hong Y, Cho JH, Choi JW, et al. Single-cell RNA sequencing demonstrates the molecular and cellular reprogramming of metastatic lung adenocarcinoma. Nat Commun. 2020;11(1):2285.
3. Laughney AM, Hu J, Campbell NR, Bakhoum SF, Setty M, Lavallee VP, et al. Regenerative lineages and immune-mediated pruning in lung cancer metastasis. Nat Med. 2020;26(2):259-69.
4. Wu F, Fan J, He Y, Xiong A, Yu J, Li Y, et al. Single-cell profiling of tumor heterogeneity and the microenvironment in advanced non-small cell lung cancer. Nat Commun. 2021;12(1):2540.
5. Xing X, Yang F, Huang Q, Guo H, Li J, Qiu M, et al. Decoding the multicellular ecosystem of lung adenocarcinoma manifested as pulmonary subsolid nodules by single-cell RNA sequencing. Sci Adv. 2021;7(5).
6. Hou J, Aerts J, den Hamer B, van Ijcken W, den Bakker M, Riegman P, et al. Gene expression-based classification of non-small cell lung carcinomas and survival prediction. PLoS One. 2010;5(4):e10312.
7. Rousseaux S, Debernardi A, Jacquiau B, Vitte AL, Vesin A, Nagy-Mignotte H, et al. Ectopic activation of germline and placental genes identifies aggressive metastasis-prone lung cancers. Sci Transl Med. 2013;5(186):186ra66.
8. Okayama H, Kohno T, Ishii Y, Shimada Y, Shiraishi K, Iwakawa R, et al. Identification of genes upregulated in ALK-positive and EGFR/KRAS/ALK-negative lung adenocarcinomas. Cancer Res. 2012;72(1):100-11.
9. Botling J, Edlund K, Lohr M, Hellwig B, Holmberg L, Lambe M, et al. Biomarker discovery in non-small cell lung cancer: integrating gene expression profiling, meta-analysis, and tissue microarray validation. Clin Cancer Res. 2013;19(1):194-204.
10. Der SD, Sykes J, Pintilie M, Zhu CQ, Strumpf D, Liu N, et al. Validation of a histology-independent prognostic gene signature for early-stage, non-small-cell lung cancer including stage IA patients. J Thorac Oncol. 2014;9(1):59-64.
11. Schabath MB, Welsh EA, Fulp WJ, Chen L, Teer JK, Thompson ZJ, et al. Differential association of STK11 and TP53 with KRAS mutation-associated gene expression, proliferation and immune surveillance in lung adenocarcinoma. Oncogene. 2016;35(24):3209-16.
12. Goldman MJ, Craft B, Hastie M, Repečka K, McDade F, Kamath A, et al. Visualizing and interpreting cancer genomics data via the Xena platform. Nat Biotechnol. 2020;38(6):675-8.
13. Xu JY, Zhang C, Wang X, Zhai L, Ma Y, Mao Y, et al. Integrative Proteomic Characterization of Human Lung Adenocarcinoma. Cell. 2020;182(1):245-61.e17.
