# Supplementary material for: UPP1 promotes lung adenocarcinoma progression through the induction of an immunosuppressive microenvironment
Source: Nat Commun. 2024 Feb 8;15:1200. doi: 10.1038/s41467-024-45340-w (PMC10853547; doi:10.1038/s41467-024-45340-w)
Supplement: Supplementary file 3 — Description of Additional Supplementary Files [file 41467_2024_45340_MOESM3_ESM.pdf]

Description of Additional Supplementary Files

File Name: Supplementary Data 1
Description: Transcriptomic and proteomic resources included in this study.

File Name: Supplementary Data 2
Description: Differential marker genes of each cell cluster (Statistical analysis was conducted using Wilcoxon Rank Sum Test with Bonferroni correction).

File Name: Supplementary Data 3
Description: Gene signatures used for functional analysis.

File Name: Supplementary Data 4
Description: Functional enrichment analysis of UPP1 tumor cell population (Statistical analysis was conducted using Wilcoxon Rank Sum Test with Bonferroni correction. Terms with adjusted p-values < 0.05 are shown).

File Name: Supplementary Data 5
Description: Clinical information of LUAD samples from Zhongshan Hospital.

File Name: Supplementary Data 6
Description: Identification of cytokines related to UPP1 up-regulation using the RayBio human cytokine antibody array.
