# Supplementary material for: UPP1 promotes lung adenocarcinoma progression through the induction of an immunosuppressive microenvironment
Source: Nat Commun. 2024 Feb 8;15:1200. doi: 10.1038/s41467-024-45340-w (PMC10853547; doi:10.1038/s41467-024-45340-w)
Supplement: Supplementary file 10 — Reporting Summary [file 41467_2024_45340_MOESM10_ESM.pdf]

nature portfolio

Corresponding author(s): Chunlai Lu
Last updated by author(s): Jan 13, 2024

Reporting Summary

Nature Portfolio wishes to improve the reproducibility of the work that we publish. This form provides structure for consistency and transparency in reporting. For further information on Nature Portfolio policies, see our Editorial Policies and the Editorial Policy Checklist.

Statistics

For all statistical analyses, confirm that the following items are present in the figure legend, table legend, main text, or Methods section.

n/a	Confirmed	
☐	☒	The exact sample size (*n*) for each experimental group/condition, given as a discrete number and unit of measurement
☐	☒	A statement on whether measurements were taken from distinct samples or whether the same sample was measured repeatedly
☐	☒	The statistical test(s) used AND whether they are one- or two-sided<br>*Only common tests should be described solely by name; describe more complex techniques in the Methods section.*
☒	☐	A description of all covariates tested
☐	☒	A description of any assumptions or corrections, such as tests of normality and adjustment for multiple comparisons
☐	☒	A full description of the statistical parameters including central tendency (e.g. means) or other basic estimates (e.g. regression coefficient) AND variation (e.g. standard deviation) or associated estimates of uncertainty (e.g. confidence intervals)
☐	☒	For null hypothesis testing, the test statistic (e.g. *F*, *t*, *r*) with confidence intervals, effect sizes, degrees of freedom and *P* value noted<br>*Give P values as exact values whenever suitable.*
☒	☐	For Bayesian analysis, information on the choice of priors and Markov chain Monte Carlo settings
☒	☐	For hierarchical and complex designs, identification of the appropriate level for tests and full reporting of outcomes
☐	☒	Estimates of effect sizes (e.g. Cohen's *d*, Pearson's *r*), indicating how they were calculated

Our web collection on statistics for biologists contains articles on many of the points above.

Software and code

Policy information about availability of computer code

Data collection	Pannoramic MIDI and Olympus VS200 for IHC and mIF; Sony MA900 Multi-Application Cell Sorter for flow cytometry and cell sorting; E-blot TOUCH IMAGER for western blotting and cytokine arrary analysis; 10X Genomics and Illumina NextSeq 2000 platform for single-cell RNA sequencing; BIO-RAD Real-time PCR System for RT-qPCR; SpectraMax ABS Plus microplate reader for cell viability and ELISA detection; CSU-W1 confocal microscopy for IF; CyTOF XT system for CyTOF.
Data analysis	The Cell Ranger toolkit (version 6.1.2) for for scRNA-seqraw data analysis; scRNA-seq data downstream analysis was conducted in R (4.2.1) using Seurat (version 4.0.5) R package; Other R packages used included: Harmony 0.1.1; singleR 2.0.0; inferCNV 1.14.0; sva 3.48.0; ESTIMATE 1.0.13; ssGSEA 1.44.0 ; AUCell1.20.2; GSVA 1.44.0; survminer 0.4.9; pRRophetic 1.0.0; CellPhoneDB 2.1.7; mIF: Visiopharm V2023.8; CyTOF: Cytobank (http://www.cytobank.cn)

For manuscripts utilizing custom algorithms or software that are central to the research but not yet described in published literature, software must be made available to editors and reviewers. We strongly encourage code deposition in a community repository (e.g. GitHub). See the Nature Portfolio guidelines for submitting code & software for further information.

Data

Policy information about availability of data

All manuscripts must include a data availability statement. This statement should provide the following information, where applicable:

- Accession codes, unique identifiers, or web links for publicly available datasets
- A description of any restrictions on data availability
- For clinical datasets or third party data, please ensure that the statement adheres to our policy

The LUAD scRNA-seq publicly available data used in this study are available in the GSA database under accession code CRA001963 (https://ngdc.cncb.ac.cn/gsa/browse/CRA001963)(80) and HRA000154 (https://ngdc.cncb.ac.cn/gsa-human/browse/HRA000154)(84), and GEO database under accession code GSE131907 (https://www.ncbi.nlm.nih.gov/geo/query/acc.cgi?acc=GSE131907)(81), GSE123904 (https://www.ncbi.nlm.nih.gov/geo/query/acc.cgi?acc=GSE123904)(82), and GSE148071 (https://www.ncbi.nlm.nih.gov/geo/query/acc.cgi?acc=GSE148071)(83). The LUAD bulk publicly available data used in this study are available in the GEO database under accession code GSE19188 (https://www.ncbi.nlm.nih.gov/geo/query/acc.cgi?acc=GSE19188)(113), GSE30219 (https://www.ncbi.nlm.nih.gov/geo/query/acc.cgi?acc=GSE30219)(114), GSE31210 (https://www.ncbi.nlm.nih.gov/geo/query/acc.cgi?acc=GSE31210)(115), GSE37745 (https://www.ncbi.nlm.nih.gov/geo/query/acc.cgi?acc=GSE37745)(116), GSE50081 (https://www.ncbi.nlm.nih.gov/geo/query/acc.cgi?acc=GSE50081)(117), and GSE72094 (https://www.ncbi.nlm.nih.gov/geo/query/acc.cgi?acc=GSE72094)(118). The TCGA publicly available data used in this study are available in the Xena database (Batch effects normalized mRNA data, Pan-Cancer Atlas Hub) (https://xenabrowser.net)(119). The scRNA-seq data generated in this study have been deposited in the GSA database under accession code HRA003967 (https://ngdc.cncb.ac.cn/gsa-human/browse/HRA003967). The remaining data are available within the Article, Supplementary Information or Source Data file. Source data are provided with this paper.

Research involving human participants, their data, or biological material

Policy information about studies with human participants or human data. See also policy information about sex, gender (identity/presentation), and sexual orientation and race, ethnicity and racism.

Reporting on sex and gender	For the TMA study, the participant count was 123 females and 82 males. In the mIF analysis, there were 8 females and 7 males. For the Organoid research, the number of participants was 3 females and 3 males. Detailed characteristics of these participants are summarized in Supplementary Data 5.
Reporting on race, ethnicity, or other socially relevant groupings	All patients included in this study are of Chinese ancestry.
Population characteristics	For TMA study, there are 123 females and 82 males with ages ranging from 27 to 83 years, with a median age of 61 and an average age of 60.45 years. In the mIF analysis, there are 8 females and 7 males. The age of the individuals ranges from 57 to 77 years, with a median age of 63, a mean age of 64.67. In organoid cohort, 3 females and 3 males with ages ranging from 58 to 71 years, and a median age 66.5 years, a mean age of 66.17.
Recruitment	Three different batches LUAD samples were collected from Zhongshan Hospital in the current study. The TMA consists of 205 LUAD patients who underwent surgical resection of pulmonary carcinoma between January 2013 and August 2013. The tumor stages were determined based on the latest edition of the American Joint Committee on Cancer (AJCC) TNM classification. The overall survival and recurrence-free survival times were tracked from the day of the resection surgery up to the day of death, relapse/metastasis, or the last follow-up in August 2018. Tissues for this cohort were preserved in FFPE blocks and were sliced and fixed on microscope slides for immunohistochemistry in this study; LUAD samples used for multiplex immunofluorescence (mIF) analysis comprises 15 LUAD samples collected from Zhongshan Hospital between 2021 and 2022. The fresh samples were fixed using 4% paraformaldehyde and later processed into paraffin blocks. Similar to the TMA cohort, tissues for this batch were preserved in FFPE blocks. These samples were then sliced and mounted onto microscope slides for the subsequent immunofluorescence analysis in this study; six LUAD samples used for the establishment of patient-derived organoids (PDOs) were collected from Zhongshan Hospital in 2022.
Ethics oversight	This study was approved by the ethics committee of Zhongshan Hospital, Fudan University (B2021-128). All participants from Zhongshan hospital donating surgical tissues provided written informed consent. All diagnoses were confirmed by histological reviews by qualified pathologists.

Note that full information on the approval of the study protocol must also be provided in the manuscript.

Field-specific reporting

Please select the one below that is the best fit for your research. If you are not sure, read the appropriate sections before making your selection.

☒ Life sciences ☐ Behavioural & social sciences ☐ Ecological, evolutionary & environmental sciences

For a reference copy of the document with all sections, see nature.com/documents/nr-reporting-summary-flat.pdf

Life sciences study design

All studies must disclose on these points even when the disclosure is negative.

Sample size	For in vitro experiments, a sample size of n≥3 biological replications was used, following standard practices in the research field. This selection

Sample size	was made to ensure reproducible results. For mouse studies, based on our extensive experience and previous literature, mice were randomly assigned to groups, each consisting of at least 4 animals, to ensure the production of reproducible results. Regarding patient samples, for tissue microarrays, our sample size was determined based on the availability of well-preserved pathological specimens with follow-up information. For multiplex immunofluorescence patient samples and organoid samples, we decided on a sample size of n≥3, following standard practices in the field.
Data exclusions	No data were excluded from analysis.
Replication	For each set of representative data, experiments were conducted a minimum of three times to ensure consistent results, except where specifically indicated otherwise in the manuscript
Randomization	Animals were randomly allocated to their respective groups. For cell culture experiments, individual wells were assigned to treatments in a random manner. For tissue microarray analysis, AI-based software was utilized for unbiased and uniform analysis, with retrospective prognostic analysis of patients based on the expression levels of target molecules. In multiplex immunofluorescence analysis, AI-based Visiopharm software was used for consistent and unbiased analysis, where Visiopharm's results automatically categorize the samples into high and low expression groups. For organoid analysis, the research aimed to determine high and low expression groups based on quantitative immunohistochemistry, without involving random grouping.
Blinding	Due to the objective nature of the molecular biological assays employed in the study, investigators were not blinded to the treatments. However, it should be noted that no subjective assessments were involved in the evaluation process.

Reporting for specific materials, systems and methods

We require information from authors about some types of materials, experimental systems and methods used in many studies. Here, indicate whether each material, system or method listed is relevant to your study. If you are not sure if a list item applies to your research, read the appropriate section before selecting a response.

Materials & experimental systems

n/a	Involved in the study
☐	☒ Antibodies
☐	☒ Eukaryotic cell lines
☒	☐ Palaeontology and archaeology
☐	☒ Animals and other organisms
☒	☐ Clinical data
☒	☐ Dual use research of concern
☒	☐ Plants

Methods

n/a	Involved in the study
☒	☐ ChIP-seq
☐	☒ Flow cytometry
☒	☐ MRI-based neuroimaging

Antibodies

Antibodies used

Antibodies:
Immunohistochemistry (IHC):
Anti-human-UPP1 (Abcam, ab185680), 1:100
Anti-human-PD-L1 (Cell Signaling Technology, 13684), 1:100, Clone E1L3N
Anti-human-p63 IHC kit (Proteintech, KHC0086), working solution
Anti-human-TTF-1 (Maixin biotech, MAB-0677), 1:100, Clone MX011
Anti-human-CK5 (Proteintech, 66727-1-Ig), 1:100, Clone 1A1C5
Anti-rabbit IgG HRP-linked-antibody (Servicebio, GB23303), 1:200

mIF:
Anti-human-CD4 (Abcam, ab133616), 1:100, Clone EPR6855
Anti-human-FOXP3 (Abcam, ab191416), 1:100, Clone EPR15038-69
Anti-human-UPP1 (Proteintech, 14186-1-AP), 1:100
Anti-human-α-SMA (Bioss, bsm-33187m), 1:500, Clone 3F9
Anti-human-MMP11 (Abcam, ab119284), 1:100
Anti-human-PD-1 (CST, 86163S), 1:100, Clone D4W2J
Anti-human-LAG3 (CST, 15372S), 1:100, Clone D2G4O
Anti-human-CD8 (Proteintech, 66868-1-Ig), 1:200, Clone 1G2B10
Anti-human-SPP1 (Proteintech, 22952-1-AP), 1:100
Anti-human-CD68 (Abcam, ab213363), 1:100, Clone EPR20545
Anti-human-PanCK (Proteintech, 26411-1-AP), 1:200
Anti-human-CK5 (Proteintech, 66727-1-Ig), 1:200, Clone 1A1C5
Anti-human-TTF-1 (Maixin biotech, MAB-0677), 1:100, Clone MX011
Anti-rabbit IgG antibody FlexAble 488 (Proteintech, KFA001), working solution
Anti-rabbit IgG antibody FlexAble 750 (Proteintech, KFA004), working solution
Anti-mouse IgG antibody FlexAble 555 (Proteintech, KFA022), working solution
Anti-rabbit IgG antibody AF555 (Abcam, ab150078), 1:1000
Anti-rabbit IgG antibody AF647 (Beyotime, A0468), 1:500
Anti-rabbit IgG antibody mCherry (Absin, abs50028), working solution

Western Blotting:
Anti-human-UPP1 (Abcam, ab128854), 1:1000, Clone EPR7680
Anti-human-PD-L1 (Cell Signaling Technology, 13684), 1:1000, Clone E1L3N
Anti-human-β-Actin (Proteintech, HRP-60008), 1:2000, Clone 7D2C10
Anti-human-SPP1 (Proteintech, 22952-1-AP), 1:1000
Anti-human-GAPDH (Proteintech, HRP-60004), 1:5000, Clone 1E6D9
Anti-human-MMP11 (Abcam, ab119284), 1:1000
Anti-human-FAP (Abcam, ab207178), 1:1000, Clone EPR20021
Anti-human-PI3K (Cell Signaling Technology, 4257S), 1:1000, Clone 19H8
Anti-human-AKT (Cell Signaling Technology, 9272S), 1:1000
Anti-human-mTOR (Cell Signaling Technology, 2972S), 1:1000
Anti-human-PI3K p85 (Abcam, ab278545), 1:1000, Clone PI3KY458-1A11
Anti-human-p-mTOR (Abcam, ab109268), 1:1000, Clone EPR426(2)
Anti-human-p-AKT (Santa Cruz, sc-293125), 1:200, Clone 5.Ser 473
Anti-rabbit IgG HRP-linked-antibody (Cell Signaling, 7074S), 1:2000
Anti-mouse IgG HRP-linked-antibody (Jackson Immunoresearch, 115-035-003E), 1:10000

Flow Cytometry:
Anti-human-CD4-FITC (Invitrogen, 11-0048-42), 1:100, clone OKT4
Anti-human-FOXP3-PE (Invitrogen, 12-4776-42), 1:20, clone PCH101
Anti-human-CD25-APC (Invitrogen, 17-0257-42), 1:20, clone CD25-4E3
Anti-human-CD8-PE (Biolegend, 344705), 1:100, clone SK1
Anti-human-LAG3-APC (Biolegend, 369211), 1:20, clone 7H2C65
Anti-human-CD279-FITC (Invitrogen, 11-9969-42), 1:50, clone MIH4
Anti-human-CD274-PE (Invitrogen, 12-5983-42), 1:200, clone MIH1
Anti-mouse-CD8-APC (Biolegend, 140410), 1:100, clone 53-5.8
Anti-mouse-Perforin-PE (Invitrogen, 12-9392-80), 1:20, clone eBioOMAK-D
Anti-mouse-Granzyme B-FITC (Biolegend, 372206), 1:20, clone QA16A02
Anti-mouse-CD45-FITC (Invitrogen, 11-0451-81), 1:100, clone 30-F11
Anti-mouse-CD140-PE (Biolegend, 135905), 1:100, clone APA5
Anti-mouse-CD31-APC (Biolegend, 102410), 1:100, clone 390
Anti-mouse-CD45-BV421 (Biolegend, 103134), 1:100, clone 30-F11
Anti-mouse-CD3-APC (Biolegend, 100326), 1:100, clone 145-2C11
Anti-mouse-CD8-BV711 (Invitrogen, 407-0081-82), 1:100, clone 53-6.7
Anti-mouse-IFN-γ-BV510 (Biolegend, 505841), 1:20, clone XMG1.2
Anti-mouse-TNF-α-BV605 (Biolegend, 506329), 1:50, clone MP6-XT22

CyTOF:
Anti-mouse-CD11b-110Cd (Fluidigm, 92J001110), 1:100, clone M1/70
Anti-mouse-TCRb-143Nd (Fluidigm, 3143010B), 1:100, clone H57-597
Anti-mouse-CD25-150Nd (Fluidigm, 3150002B), 1:100, clone 3C7
Anti-mouse-CD3e-152Sm (Fluidigm, 3152004B), 1:100, clone 145-2C11
Anti-mouse-PD-L1-153Eu (Fluidigm, 3153031B), 1:100, clone MIH5
Anti-mouse-PD-1-159Tb (Fluidigm, 3159006B), 1:100, clone RMP1-30
Anti-mouse-CD163-161Dy-self-conjugate (Abcam, ab213612), 1:100, clone EPR19518
Anti-mouse-TIM3-162Dy (Fluidigm, 3162029B), 1:100, clone RMT3-23
Anti-mouse-CD8a-168Er (Fluidigm, 3168003B), 1:200, clone 53-6.7
Anti-mouse-NK1.1-170Er (Fluidigm, 3170002B), 1:100, clone PK136
Anti-mouse-CD19-171Yb (Fluidigm, 92J025171), 1:200, clone 6D5
Anti-mouse-CD4-172Yb (Fluidigm, 3172003B), 1:200, clone RM4-5
Anti-mouse-LAG3-174Yb (Fluidigm, 3174019B), 1:100, clone C9B7W
Anti-mouse-CTLA-4-175Lu-self-conjugate (Abcam, ab251599), 1:100, clone CAL49
Anti-mouse-Ly-6G-195Pt (Fluidigm, 92J011195), 1:200, clone 1A8
Anti-mouse-CD45-89Y (Fluidigm, 3089005B), 1:200, clone 30-F11
Anti-mouse-TNF-α-141Pr (Fluidigm, 3141013B), 1:100, clone MP6-XT22
Anti-mouse-IL-4-155Gd-self-conjugate (Biolegend, 504129), 1:100, clone 11B11
Anti-mouse-IL-10-158Gd (Fluidigm, 3158002B), 1:100, clone JES5-16E3
Anti-mouse-IFNg-165Ho (Fluidigm, 3165003B), 1:100, clone XMG1.2
Anti-mouse-FOXP3-149Sm-self-conjugate (Invitrogen, 14-5773-82), 1:100, clone FJK-16s

Validation

Antibodies:
Immunohistochemistry (IHC):
Anti-human-UPP1 (Abcam, ab185680), Supplier validation: "Reacts with: Human, applications Suitable for: IHC, IF".
Anti-human-PD-L1 (Cell Signaling Technology, 13684), Supplier validation: "Reacts with: Human, applications Suitable for: IHC, WB, IP".
Anti-human-p63 IHC kit (Proteintech, KHC0086), Supplier validation: "Reacts with: Human, mouse, rat, applications Suitable for: IHC ".
Anti-human-TTF-1 (Maixin biotech, MAB-0677), Supplier validation: "Reacts with: Human, applications Suitable for: IHC ".
Anti-human-CK5 (Proteintech, 66727-1-Ig), Supplier validation: "Reacts with: Human, mouse, rat, applications Suitable for: IHC ".
Anti-rabbit IgG HRP-linked-antibody (Servicebio, GB23303), Supplier validation: "Reacts with: Rabbit, applications Suitable for: IHC, WB, ELISA".

mIF:
Anti-human-CD4 (Abcam, ab133616), Supplier validation: "Reacts with: Human, applications Suitable for: WB, IHC, IF".
Anti-human-FOXP3 (Abcam, ab191416), Supplier validation: "Reacts with: Human, applications Suitable for: WB, IHC ".

Anti-human-UPP1 (Proteintech, 14186-1-AP), Supplier validation: "Reacts with: Human, mouse, applications Suitable for: IHC, WB, ELISA ".
Anti-human-α-SMA (Bioss, bsm-33187m), Supplier validation: "Reacts with: Human, mouse, rat, applications Suitable for: WB, IHC, IF ".
Anti-human-MMP11 (Abcam, ab119284), Supplier validation: "Reacts with: Human, applications Suitable for: WB, IHC, IF ".
Anti-human-PD-1 (CST, 86163S), Supplier validation: "Reacts with: Human, applications Suitable for: WB, IHC, IF ".
Anti-human-LAG3 (CST, 15372S), Supplier validation: "Reacts with: Human, applications Suitable for: WB, IHC ".
Anti-human-CD8 (Proteintech, 66868-1-Ig), Supplier validation: "Reacts with: Human, applications Suitable for: WB, IHC, IF ".
Anti-human-SPP1 (Proteintech, 22952-1-AP), Supplier validation: "Reacts with: Human, mouse, rat, applications Suitable for: WB, IHC, IF ".
Anti-human-CD68 (Abcam, ab213363), Supplier validation: "Reacts with: Human, applications Suitable for: WB, IHC, IF ".
Anti-human-PanCK (Proteintech, 26411-1-AP), Supplier validation: "Reacts with: Human, applications Suitable for: WB, IHC, IF ".
Anti-human-CK5 (Proteintech, 66727-1-Ig), Supplier validation: "Reacts with: Human, mouse, applications Suitable for: WB, IHC, IF ".
Anti-human-TTF-1 (Maixin biotech, MAB-0677), Supplier validation: "Reacts with: Human, applications Suitable for: IHC, IF ".
Anti-rabbit IgG antibody FlexAble 488 (Proteintech, KFA001), Supplier validation: "Reacts with: Rabbit, applications Suitable for: WB, IF, FC ".
Anti-rabbit IgG antibody FlexAble 750 (Proteintech, KFA004), Supplier validation: "Reacts with: Rabbit, applications Suitable for: WB, IF, FC ".
Anti-mouse IgG antibody FlexAble 555 (Proteintech, KFA022), Supplier validation: "Reacts with: Mouse, applications Suitable for: WB, IF, FC ".
Anti-rabbit IgG antibody AF555 (Abcam, ab150078), Supplier validation: "Reacts with: Rabbit, applications Suitable for: IHC, IF, FC ".
Anti-rabbit IgG antibody AF647 (Beyotime, A0468), Supplier validation: "Reacts with: Rabbit, applications Suitable for: IF ".
Anti-rabbit IgG antibody mCherry (Absin, abs50028), Supplier validation: "Reacts with: Rabbit, applications Suitable for: IF ".

Western Blotting:
Anti-human-UPP1 (Abcam, ab128854), Supplier validation: "Reacts with: Human, applications Suitable for: WB ".
Anti-human-PD-L1 (Cell Signaling Technology, 13684), Supplier validation: "Reacts with: Human, applications Suitable for: IHC, WB, IP".
Anti-human-β-Actin (Proteintech, HRP-60008), Supplier validation: "Reacts with: Human, Mouse, Rat, applications Suitable for: WB ".
Anti-human-SPP1 (Proteintech, 22952-1-AP), Supplier validation: "Reacts with: Human, mouse, rat, applications Suitable for: WB, IHC, IF ".
Anti-human-GAPDH (Proteintech, HRP-60004), Supplier validation: "Reacts with: Human, Mouse, Rat, applications Suitable for: WB ".
Anti-human-MMP11 (Abcam, ab119284), Supplier validation: "Reacts with: Human, applications Suitable for: WB, IHC, IF ".
Anti-human-FAP (Abcam, ab207178), Supplier validation: "Reacts with: Human, applications Suitable for: WB, IHC ".
Anti-human-PI3K (Cell Signaling Technology, 4257S), Supplier validation: "Reacts with: Human, mouse, rat, applications Suitable for: WB, IP ".
Anti-human-AKT (Cell Signaling Technology, 9272S), Supplier validation: "Reacts with: Human, mouse, rat, applications Suitable for: WB, IP, IF ".
Anti-human-mTOR (Cell Signaling Technology, 2972S), Supplier validation: "Reacts with: Human, mouse, rat, applications Suitable for: WB, IP ".
Anti-human-PI3K p85 (Abcam, ab278545), Supplier validation: "Reacts with: Human, mouse, applications Suitable for: WB, FC ".
Anti-human-p-mTOR (Abcam, ab109268), Supplier validation: "Reacts with: Human, mouse, applications Suitable for: WB, IHC ".
Anti-human-p-AKT (Santa Cruz, sc-293125), Supplier validation: "Reacts with: Human, mouse, rat, applications Suitable for: WB, IHC ".
Anti-rabbit IgG HRP-linked-antibody (Cell Signaling, 7074S), Supplier validation: "Reacts with: Rabbit, applications Suitable for: WB ".
Anti-mouse IgG HRP-linked-antibody (Jackson Immunoresearch, 115-035-003E), Supplier validation: "Reacts with: Mouse, applications Suitable for: WB, IHC, ELISA ".

Flow Cytometry:
Anti-human-CD4-FITC (Invitrogen, 11-0048-42), Supplier validation: "Reacts with: Human, applications Suitable for: FC, IF ".
Anti-human-FOXP3-PE (Invitrogen, 12-4776-42), Supplier validation: "Reacts with: Human, applications Suitable for: FC, IF ".
Anti-human-CD25-APC (Invitrogen, 17-0257-42), Supplier validation: "Reacts with: Human, applications Suitable for: FC ".
Anti-human-CD8-PE (Biolegend, 344705), Supplier validation: "Reacts with: Human, applications Suitable for: FC ".
Anti-human-LAG3-APC (Biolegend, 369211), Supplier validation: "Reacts with: Human, applications Suitable for: FC ".
Anti-human-CD279-FITC (Invitrogen, 11-9969-42), Supplier validation: "Reacts with: Human, applications Suitable for: FC, IHC ".
Anti-human-CD274-PE (Invitrogen, 12-5983-42), Supplier validation: "Reacts with: Human, applications Suitable for: FC, IHC ".
Anti-mouse-CD8-APC (Biolegend, 140410), Supplier validation: "Reacts with: Mouse, applications Suitable for: FC ".
Anti-mouse-Perforin-PE (Invitrogen, 12-9392-80), Supplier validation: "Reacts with: Mouse, applications Suitable for: FC ".
Anti-mouse-Granzyme B-FITC (Biolegend, 372206), Supplier validation: "Reacts with: Human, mouse, applications Suitable for: FC ".
Anti-mouse-CD45-FITC (Invitrogen, 11-0451-81), Supplier validation: "Reacts with: Mouse, applications Suitable for: FC, IHC, IF ".
Anti-mouse-CD140-PE (Biolegend, 135905), Supplier validation: "Reacts with: Mouse, applications Suitable for: FC ".
Anti-mouse-CD31-APC (Biolegend, 102410), Supplier validation: "Reacts with: Mouse, applications Suitable for: FC ".
Anti-mouse-CD45-BV421 (Biolegend, 103134), Supplier validation: "Reacts with: Mouse, applications Suitable for: FC ".
Anti- mouse-CD3-APC (Biolegend, 100326), Supplier validation: "Reacts with: Mouse, applications Suitable for: FC ".
Anti-mouse-CD8-BV711 (Invitrogen, 407-0081-82), Supplier validation: "Reacts with: Mouse, applications Suitable for: FC ".
Anti-mouse-IFN-γ-BV510 (Biolegend, 505841), Supplier validation: "Reacts with: Mouse, applications Suitable for: FC ".
Anti-mouse-TNF-α-BV605 (Biolegend, 506329), Supplier validation: "Reacts with: Mouse, applications Suitable for: FC ".

CyTOF:
Anti-mouse-CD11b-110Cd (Fluidigm, 92J001110), Supplier validation: "Reacts with: Mouse, applications Suitable for: CYTOF ".
Anti-mouse-TCRb-143Nd (Fluidigm, 3143010B), Supplier validation: "Reacts with: Mouse, applications Suitable for: CYTOF ".
Anti-mouse-CD25-150Nd (Fluidigm, 3150002B), Supplier validation: "Reacts with: Mouse, applications Suitable for: CYTOF ".
Anti-mouse-CD3e-152Sm (Fluidigm, 3152004B), Supplier validation: "Reacts with: Mouse, applications Suitable for: CYTOF ".
Anti-mouse-PD-L1-153Eu (Fluidigm, 3153031B), Supplier validation: "Reacts with: Mouse, applications Suitable for: CYTOF ".
Anti-mouse-PD-1-159Tb (Fluidigm, 3159006B), Supplier validation: "Reacts with: Mouse, applications Suitable for: CYTOF ".
Anti-mouse-CD163-161Dy-self-conjugate (Abcam, ab213612), Supplier validation: "Reacts with: Mouse, human, applications Suitable for: WB, IHC, FC ".

Anti-mouse-TIM3-162Dy (Fluidigm, 3162029B), Supplier validation: "Reacts with: Mouse, applications Suitable for: CYTOF ".
Anti-mouse-CD8a-168Er (Fluidigm, 3168003B), Supplier validation: "Reacts with: Mouse, applications Suitable for: CYTOF ".
Anti-mouse-NK1.1-170Er (Fluidigm, 3170002B), Supplier validation: "Reacts with: Mouse, applications Suitable for: CYTOF ".
Anti-mouse-CD19-171Yb (Fluidigm, 92J025171), Supplier validation: "Reacts with: Mouse, applications Suitable for: CYTOF ".
Anti-mouse-CD4-172Yb (Fluidigm, 3172003B), Supplier validation: "Reacts with: Mouse, applications Suitable for: CYTOF ".
Anti-mouse-LAG3-174Yb (Fluidigm, 3174019B), Supplier validation: "Reacts with: Mouse, applications Suitable for: CYTOF ".
Anti-mouse-CTLA-4-175Lu-self-conjugate (Abcam, ab251599), Supplier validation: "Reacts with: Mouse, Human, applications Suitable for: IHC, FC, WB ".
Anti-mouse-Ly-6G-195Pt (Fluidigm, 92J011195), Supplier validation: "Reacts with: Mouse, applications Suitable for: CYTOF ".
Anti-mouse-CD45-89Y (Fluidigm, 3089005B), Supplier validation: "Reacts with: Mouse, applications Suitable for: CYTOF ".
Anti-mouse-TNF-α-141Pr (Fluidigm, 3141013B), Supplier validation: "Reacts with: Mouse, applications Suitable for: CYTOF ".
Anti-mouse-IL-4-155Gd-self-conjugate (Biolegend, 504129), Supplier validation: "Reacts with: Mouse, applications Suitable for: CYTOF ".
Anti-mouse-IL-10-158Gd (Fluidigm, 3158002B), Supplier validation: "Reacts with: Mouse, applications Suitable for: CYTOF ".
Anti-mouse-IFNg-165Ho (Fluidigm, 3165003B), Supplier validation: "Reacts with: Mouse, applications Suitable for: CYTOF ".
Anti-mouse-FOXP3-149Sm-self-conjugate (Invitrogen, 14-5773-82), Supplier validation: "Reacts with: Mouse, applications Suitable for: FC, IHC, IF ".

Eukaryotic cell lines

Policy information about cell lines and Sex and Gender in Research

Cell line source(s)	The cell lines HCC827 (CL-0094), LLC (CL-0140), HFL1 (CL-0106), and THP-1 (CL-0233) were purchased from Wuhan Procell Life Science and Technology Co., Ltd. (Wuhan, China). LLC-OVA cells (labeled with mCherry) were generously provided by Professor Guangchuan Wang (State Key Laboratory of Molecular Biology, Shanghai Institute of Biochemistry and Cell Biology, Center for Excellence in Molecular Cell Science, Chinese Academy of Sciences).
Authentication	STR authentication of cell lines was performed by vendors, HCC827, authenticated in October 2021; LLC, authenticated in October 2023; HFL1, authenticated in February 2023; THP-1, authenticated in February 2023
Mycoplasma contamination	Cell line was tested negative for mycoplasma contamination.
Commonly misidentified lines (See ICLAC register)	No commonly misidentified cell lines were used.

Animals and other research organisms

Policy information about studies involving animals; ARRIVE guidelines recommended for reporting animal research, and Sex and Gender in Research

Laboratory animals	Six-week-old female C57BL/6 and BALB/c nude mice were purchased from SPF Biotechnology Co., Ltd. (Beijing, China). Six-week-old female OT-1 mice were purchased from Cyagen Biosciences (Guangzhou, China). The mice were maintained in a specific-pathogen-free (SPF) environment, under a 12-hour light/dark cycle, with a temperature maintained between 22-26 degrees Celsius and a humidity level of 55±5%.
Wild animals	No wild animals were included.
Reporting on sex	Previous research has indicated that there are no significant differences between male and female mice in the LLC cell tumor model (Moreo et al. Nature Communications, 2023). During our preliminary experiments, we observed that male mice tended to engage in aggressive behavior post-transplantation, leading to the disruption of tumor growth due to fighting-related injuries. Female mice generally exhibit reduced fighting behaviors (Chaube et al. Nature Communications, 2023), which facilitates group housing and randomization, and helps in generating more consistent results, so female mice were used.
Field-collected samples	No field-collected samples were used in this study.
Ethics oversight	All animal experiments were approved by the Ethics Committee of Zhongshan Hospital, Fudan University.

Note that full information on the approval of the study protocol must also be provided in the manuscript.

Plants

Seed stocks	*Report on the source of all seed stocks or other plant material used. If applicable, state the seed stock centre and catalogue number. If plant specimens were collected from the field, describe the collection location, date and sampling procedures.*
Novel plant genotypes	*Describe the methods by which all novel plant genotypes were produced. This includes those generated by transgenic approaches, gene editing, chemical/radiation-based mutagenesis and hybridization. For transgenic lines, describe the transformation method, the number of independent lines analyzed and the generation upon which experiments were performed. For gene-edited lines, describe the editor used, the endogenous sequence targeted for editing, the targeting guide RNA sequence (if applicable) and how the editor was applied.*
Authentication	*Describe any authentication procedures for each seed stock used or novel genotype generated. Describe any experiments used to assess the effect of a mutation and, where applicable, how potential secondary effects (e.g. second site T-DNA insertions, mosiacism, off-target gene editing) were examined.*

Flow Cytometry

Plots

Confirm that:

☒ The axis labels state the marker and fluorochrome used (e.g. CD4-FITC).

☒ The axis scales are clearly visible. Include numbers along axes only for bottom left plot of group (a 'group' is an analysis of identical markers).

☒ All plots are contour plots with outliers or pseudocolor plots.

☒ A numerical value for number of cells or percentage (with statistics) is provided.

Methodology

Sample preparation	For flow cytometry analysis of cell samples, single-cell suspensions were prepared in PBS and blocked using Human TruStain Fc (Fc Receptor Blocking Solution) (Biolegend, 422302) or Mouse TruStain Fc PLUS (anti-mouse CD16/32) Antibody (Biolegend, 156604). For membrane antibody staining, the antibody mixture was added into the cell suspension and incubated at room temperature for 30 minutes. The cells were then washed with cold PBS and 5 µl of 7-AAD (Biolegend, 420403) was added. These prepared samples were then subjected to flow cytometry analysis.<br>For intracellular staining, surface marker staining was first performed at room temperature for 30 minutes. Subsequently, the cells were fixed and permeabilized using the FIX & PERM Cell Permeabilization Kit (Invitrogen, GAS003). Then, intracellular antibody staining was conducted at room temperature for 2 hours. The cells were then washed with cold PBS and subjected to flow cytometry analysis. For Foxp3 staining of Tregs, we used the eBioscience Foxp3 / Transcription Factor Staining Buffer Set (Invitrogen, 00-5523-00).<br>For flow cytometry analysis of mouse tumor tissue samples, the harvested tumors were first minced. Next, they were digested using RPMI 1640 medium supplemented with 1mg/ml collagenase IV (Worthington, LS004188, for immune cell analysis) or collagenase II (Worthington, LS004176, for fibroblast analysis), and 10ug/ml DNase I (Roche, 11284932001), at 37°C on a shaking platform operating at 120 rpm. Then, the cell suspensions were passed through 70[illegible]m cell strainers to prepare single-cell suspensions in PBS. They were then blocked using Mouse TruStain Fc PLUS (anti-mouse CD16/32) Antibody (Biolegend, 156604), and the staining process was conducted as per the aforementioned procedure.
Instrument	SONY MA900 Multi-Application Cell Sorter.
Software	FlowJo software version 10.4
Cell population abundance	Cell population was gated according to its accumulation and FMO.
Gating strategy	Cells were gated by FSC/SSC to exclude debris, followed by gating FSC-A and FSC-H to separate single cells from aggregates. Detailed gating strategy shown in supplementary figures.

☒ Tick this box to confirm that a figure exemplifying the gating strategy is provided in the Supplementary Information.
